# The role of kinetic context in apparent biased agonism at GPCRs

**DOI:** 10.1038/ncomms10842

**Published:** 2016-02-24

**Authors:** Carmen Klein Herenbrink, David A. Sykes, Prashant Donthamsetti, Meritxell Canals, Thomas Coudrat, Jeremy Shonberg, Peter J. Scammells, Ben Capuano, Patrick M. Sexton, Steven J. Charlton, Jonathan A. Javitch, Arthur Christopoulos, J. Robert Lane

**Affiliations:** 1 Drug Discovery Biology, Monash Institute of Pharmaceutical Sciences, Monash University, 381 Royal Parade, Parkville, Victoria 3052, Australia; 2 Cell Signalling Research Group, School of Life Sciences, University of Nottingham, Queen's Medical Centre, Nottingham NG7 2UH, UK; 3 Departments of Psychiatry, College of Physicians and Surgeons, Columbia University, New York, New York 10032, USA; 4 Department of Pharmacology, College of Physicians and Surgeons, Columbia University, New York, New York 10032, USA; 5 Department of Medicinal Chemistry, Monash Institute of Pharmaceutical Sciences, Monash University, 381 Royal Parade, Parkville, Victoria 3052, Australia; 6 Division of Molecular Therapeutics, New York State Psychiatric Institute, New York, New York 10032, USA

## Abstract

Biased agonism describes the ability of ligands to stabilize different conformations of a GPCR linked to distinct functional outcomes and offers the prospect of designing pathway-specific drugs that avoid on-target side effects. This mechanism is usually inferred from pharmacological data with the assumption that the confounding influences of observational (that is, assay dependent) and system (that is, cell background dependent) bias are excluded by experimental design and analysis. Here we reveal that ‘kinetic context', as determined by ligand-binding kinetics and the temporal pattern of receptor-signalling processes, can have a profound influence on the apparent bias of a series of agonists for the dopamine D_2_ receptor and can even lead to reversals in the direction of bias. We propose that kinetic context must be acknowledged in the design and interpretation of studies of biased agonism.

## Abstract (web summary)

Biased agonists act at a receptor to preferentially induce distinct intracellular signalling responses over others. Here the authors show how kinetics of ligand binding and signaling responses greatly influence observed bias profiles, and hence must be considered when studying biased agonists.

## Graphical abstract

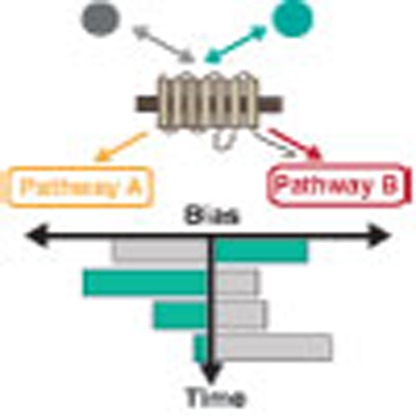

In recent years, the phenomenon of ‘biased agonism' has evolved from a theoretical concept of drug–receptor interaction to an established paradigm that is having a major impact on drug discovery^1^,^2^. Biased agonism describes the ability of different ligands to stabilize distinct conformations of a given receptor such that only a subset of the possible signalling repertoires mediated by the receptor are engaged to the relative exclusion of others^3^. To date, biased agonism has been intensively investigated in studies of G protein-coupled receptors (GPCRs), the largest class of drug targets^4^, but it is likely to be a more universal paradigm.

The promise of biased agonism is the ability to design ligands that selectively engage therapeutically relevant signalling pathways, while sparing those that contribute to undesirable side effects mediated by the same target. Indeed, biased ligands are currently being pursued in clinical trials for a number of applications^5^. Biased agonism may also explain why some drugs from a common target class (for example, β-blockers) display clinical efficacy (for example, in heart failure), whereas others fail, despite showing similar degrees of efficacy in particular preclinical indices of biological activity^6^,^7^,^8^. As the mechanistic link between cellular measures of drug–receptor efficacy and their role *in vivo* remains ill defined for many diseases, preclinical studies often focus on pathways that are ultimately not associated with the pathophysiology under investigation; failure to appreciate and capture biased agonism in such circumstances can lead to costly translational failures.

Recent studies have focused on various means for detecting biased agonists and quantifying their behaviour to inform structure–activity studies. For predictive and translational purposes, a critical aspect of any quantitative analysis of bias is the need to remove the influence of the cellular background (that is, ‘system bias' as reflected by effector–transducer complement, stoichiometry, coupling efficiency and so on) and the assay conditions (that is, ‘observational bias') when comparing the activity of a group of agonists between pathways in the same cell type^2^,^3^,^9^. In this regard, a number of analytical methods have been developed that build on the seminal operational model of agonism proposed by Black and Leff^9^,^10^,^11^,^12^. The key drug–receptor parameters in these models are the equilibrium dissociation constant of the agonist for the receptor (*K*_A_) and an operational measure of signalling efficacy (*τ*) that subsumes both receptor density and coupling efficiency to a given pathway. The *τ*/*K*_A_ ratio (‘transducer ratio') can thus be used as an index of the intrinsic relative activity of an agonist at a given pathway and represents a starting point for quantification of biased agonism. Normalizing this value for a test agonist relative to a reference agonist has been proposed to nullify the impact of system and observational bias^10^.

An example of a prototypical GPCR at which biased agonism has been extensively studied is the dopamine D_2_ receptor (D_2_R), an important therapeutic target for the treatment of neuropsychiatric disorders, including schizophrenia^13^. Prior evidence suggests that biased agonism at the D_2_R may be important for the antipsychotic activity of the partial agonist, aripiprazole^14^,^15^,^16^,^17^. However, a clear relationship between biased agonism at the D_2_R and antipsychotic efficacy remains elusive, not least due to discrepancies in the literature. For example, a recent study demonstrated that potent β-arrestin-biased D_2_R agonists were effective in preclinical animal models of psychosis^15^; however, earlier studies suggested that antagonism of β-arrestin recruitment is required for antipsychotic efficacy^16^. Our own work revealed that aripiprazole is biased towards the inhibition of cyclic AMP production as compared with ERK1/2 phosphorylation; however, this finding was inconsistent with an earlier study^17^,^18^.

It is likely to be that some of the discrepancies between studies of bias for the same ligand–receptor pairings are due to a failure to appreciate the impact of system or observational bias. In particular, the timescales of measurement of receptor signalling can vary greatly depending on the pathways under investigation and the type of readout^19^; however, all receptor models for quantifying bias assume an equilibrium. Thus, the spatiotemporal pattern of agonist action may be quite different depending on agonist-binding kinetics or if agonists differentially engage dynamic signalling and regulatory processes. Indeed, the influence of kinetics on measured drug response has long been acknowledged in the study of enzymes and receptors^20^. However, such spatiotemporal patterns of drug action may have a profound influence on the detection and interpretation of biased agonism, in particular when comparing transient responses with those measured over longer time scales. This also raises the possibility that the observed bias of a ligand may depend on the timescale(s) over which its effect is measured at different pathways.

This study is the first to systematically explore the influence of ‘kinetic context', as reflected by system and observational bias, on selected D_2_R agonists at multiple signalling pathways. We demonstrate that the major contributor to context-dependent changes in the observed bias profile of D_2_R ligands is the interplay between differential ligand-binding kinetics and the kinetics associated with different cell signalling processes, in some instances leading to complete reversals of agonist bias over time. These findings have major implications for the identification, quantification and interpretation of biased agonism at GPCRs.

## Results

### Detection of biased agonism at the D_2_R

**Figure 1:**
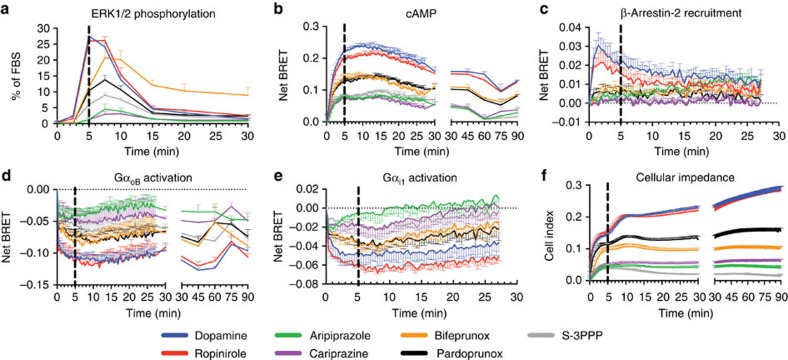
Kinetic traces of the cellular response on activation of the D_2L_R with various agonists. The effect of dopamine (10 μM), ropinirole (10 μM), aripiprazole (10 μM), cariprazine (1 μM), bifeprunox (10 μM), pardoprunox (10 μM) and S-3PPP (10 μM) on ERK1/2 phosphorylation (**a**), the inhibition of forskolin-induced cAMP production (**b**), β-arrestin-2 recruitment (**c**), activation of Gα_oB_ (**d**) and Gα_i1_ (**e**) G proteins, and CI (**f**) is shown for a period of 30–90 min. The data points are expressed as mean±s.e.m. from three (cAMP, Gα_oB_, Gα_i1_ and β-arrestin-2 recruitment) or four (ERK1/2 phosphorylation and CI) experiments performed in duplicate. Duplicates were averaged before calculating s.e.m. The raw data are included as Supplementary Data 1.

The D_2_R partial agonists selected for this study were (S)-(-)-3-(3-hydroxyphenyl)-N-propylpiperidine (S-3PPP), bifeprunox, cariprazine, aripiprazole and pardoprunox (Supplementary Fig. 1). Aripiprazole and cariprazine are D_2_R partial agonists approved as antipsychotics, whereas S-3PPP and bifeprunox were withdrawn from clinical trials due to limited antipsychotic efficacy^21^,^22^,^23^. Pardoprunox has undergone clinical trials for the treatment of Parkinson's disease. The high efficacy agonist ropinirole (used clinically to treat Parkinson's disease) and the endogenous agonist dopamine were used as reference ligands^24^. The action of these agonists was assessed at the following signalling endpoints using Flp-In-CHO cells stably expressing the long isoform of the D_2_R (Flp-In-CHO-D_2L_R), ERK1/2 phosphorylation (pERK1/2; Fig. 1a and Supplementary Data 1), inhibition of forskolin-induced cAMP production (Fig. 1b) and β-arrestin-2 recruitment (Fig. 1c). In addition, we measured activation of both Gα_i1_ and Gα_oB_ G proteins using bioluminescence resonance energy transfer (BRET)-based biosensors, as previous studies have suggested that dopamine receptor ligands differentially activate these G proteins (Fig. 1d,e)^25^,^26^. The xCELLigence system was used to evaluate the effect of the agonists on cellular impedance (CI) as a more ‘global' readout that can capture multiple convergent signalling events triggered on receptor activation (Fig. 1f)^27^. The most transient signalling response was pERK1/2 with maximal stimulation of pERK1/2 by 10 μM dopamine occurring at 5 min before a rapid return to near-basal levels after 15 min (Fig. 1a). Therefore, we first chose a 5-min time window for all assays, to compare bias at a single time point.

**Figure 2:**
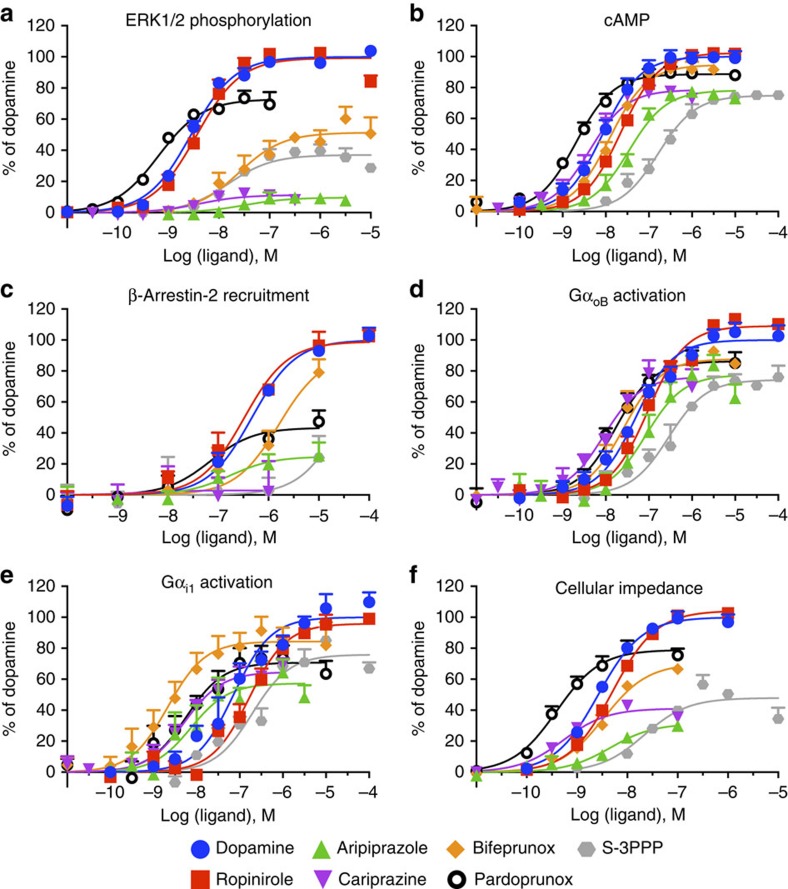
Characterization of D_2_R agonists at a range of signalling endpoints after 5 min of agonist stimulation. The ability of increasing concentrations of D_2_R agonists to induce ERK1/2 phosphorylation (**a**), inhibit 10 μM forskolin-induced cAMP production (**b**), induce β-arrestin2 recruitment (**c**), activate Gα_oB_ (**d**) and Gα_i1_ (**e**) G proteins and induce changes in CI (**f**) in Flp-In-CHO cells expressing the D_2L_R. The data were normalized to the maximal response induced by dopamine and fitted using an operational model of agonism (equation (2)). The values are expressed as mean±s.e.m. of three experiments performed in duplicate; duplicates were averaged before calculating s.e.m.

**Figure 3:**
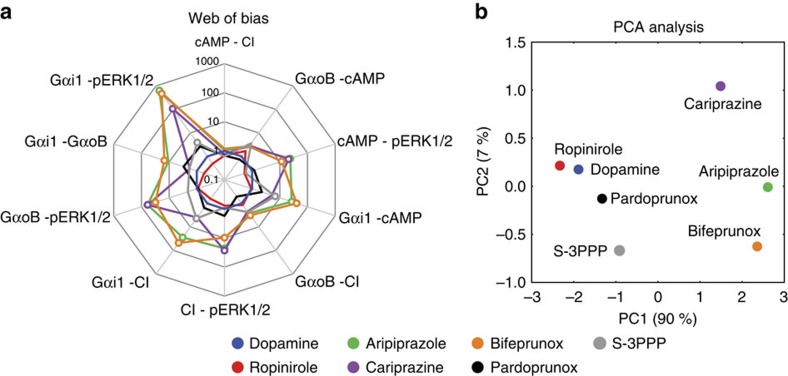
Quantification of biased agonism relative to dopamine at a 5-min time point reveals a distinct pattern of bias for D_2_R partial agonists. The concentration-response curves for various dopaminergic ligands at five different signalling endpoints were analysed using an operational model of agonism to obtain transduction coefficients (Log(*τ*/*K*_A_); Supplementary Table 1). These were normalized to the reference agonist dopamine (ΔLog(*τ*/*K*_A_); Supplementary Table 2) and then values obtained for one agonist at two different pathways were subtracted to obtain LogBias values (ΔΔLog(*τ*/*K*_A_); Supplementary Table 3). (**a**) Bias values (10^LogBias^) obtained for the agonists between the inhibition of forskolin-induced cAMP production, ERK1/2 phosphorylation, G protein activation and induction of changes in CI presented in Supplementary Table 3 are shown in a web of bias. Open circles indicate significant differences between values of ΔLog(*τ*/*K*_A_) determined at different pathways for a particular ligand, determined by a one-way analysis of variance (ANOVA) with a Tukey's post test (*P*<0.05). (**b**) A principal component analysis plot projecting the relative positions of the ligands according to the LogBias values onto the first two principal components (PC1, 90% and PC2, 7%).

Using concentration–response data obtained at 5 min for all assays (Fig. 2a–f), to determine whether the different D_2_R agonists display biased agonism, we used the operational model to derive a transduction coefficient (*τ*/*K*_A_) for each agonist at each pathway (Supplementary Table 1)^10^. The log(*τ*/*K*_A_) values were normalized to that of dopamine at each pathway (Δlog(*τ*/*K*_A_); Supplementary Table 2) and compared across different signalling pathways to give a ΔΔlog(*τ*/*K*_A_) or LogBias value (Supplementary Table 3)^10^. The Bias (antilog) values were graphically represented in a web, to illustrate a specific bias ‘fingerprint' for each individual ligand (Fig. 3a). The agonists pardoprunox and ropinirole had a similar pattern of bias to that of dopamine. S-3PPP displayed moderate (less than tenfold) bias between the activation of Gα_i1_ and all other assays apart from the activation of Gα_oB_ where there was no significant bias. Bifeprunox, aripiprazole and cariprazine had a bias fingerprint distinct from that of dopamine and the other agonists predominantly due to lower efficacy in ERK1/2 phosphorylation as compared with other signalling assays. In addition, bifeprunox and aripiprazole were significantly (one-way analysis of variance with Tukey's post test, *P*<0.05) more biased as compared with dopamine towards the activation of Gα_i1_ proteins compared with CI and inhibition of cAMP production. Bifeprunox was the only compound to display significant bias between the activation of Gα_i1_ versus Gα_oB_ G proteins. None of the agonists displayed bias between inhibition of cAMP production and the CI response. As S-3PPP and cariprazine did not give a complete concentration–response curve for β-arrestin-2 recruitment, we could not quantify their action at this pathway. Therefore, we have not included β-arrestin-2 recruitment in the web of bias but rather displayed the bias factors of the agonists between β-arrestin-2 recruitment and other signalling pathways in Supplementary Fig. 2 and Supplementary Table 4. Only bifeprunox displayed bias towards the activation of both Gα_i1_ and Gα_oB_ G proteins versus β-arrestin-2 recruitment and bias towards β-arrestin-2 recruitment versus ERK1/2 phosphorylation.

Our comparisons of the action of different ligands across multiple pathways are intrinsically multi-dimensional. We made use of principal component analysis (PCA) to distil this multi-dimensional data set into principal components, to allow us to cluster ligands into distinct bias groupings relative to dopamine (Fig. 3b)^28^. In this analysis, PC1 represents 90% of the variance and clearly demonstrates that the ligands can be subdivided into two groups based on their bias profile, with ropinirole, dopamine, S-3PPP and pardoprunox in one group and aripiprazole, bifeprunox and cariprazine in another (Fig. 3b). PC2 represents an additional 7% of the variance and further separates S-3PPP and cariprazine from their respective groups. Therefore, our quantitative approach to determining bias allowed the statistical clustering of ligands into distinct bias groupings that encompass their action across multiple pathways.

### Biased agonists display slower dissociation rates

Most measurements of receptor function are made under non-equilibrium conditions where the receptor occupancy of a ligand may differ depending on the time at which the measurement is made. This in turn may affect agonist potency^29^ and, if there are marked differences in binding kinetics within a subset of ligands, this could be interpreted as biased agonism. However, to our knowledge, the impact of binding kinetics on biased agonism has not yet been examined experimentally.

To determine the kinetic binding parameters of our selected D_2_R ligands, we conducted competition association binding experiments using membranes of Flp-In-CHO-D_2L_R and the antagonist [^3^H]spiperone^30^,^31^,^32^ in the presence of 100 μM Gpp(NH)p, to ensure that we were measuring ligand binding only to the G protein-uncoupled receptor state^33^. Although we were able to determine values of association (*k*_on_) and dissociation (*k*_off_) rate constants for the ligands that displayed a distinct bias profile at the 5-min time point (cariprazine, bifeprunox and aripiprazole), we could not do so for dopamine, ropinirole, pardoprunox and S-3PPP (Supplementary Table 5), most probably because the dissociation rate of [^3^H]spiperone (0.026 min^−1^) is slow relative to that of these four agonists.

**Figure 4:**
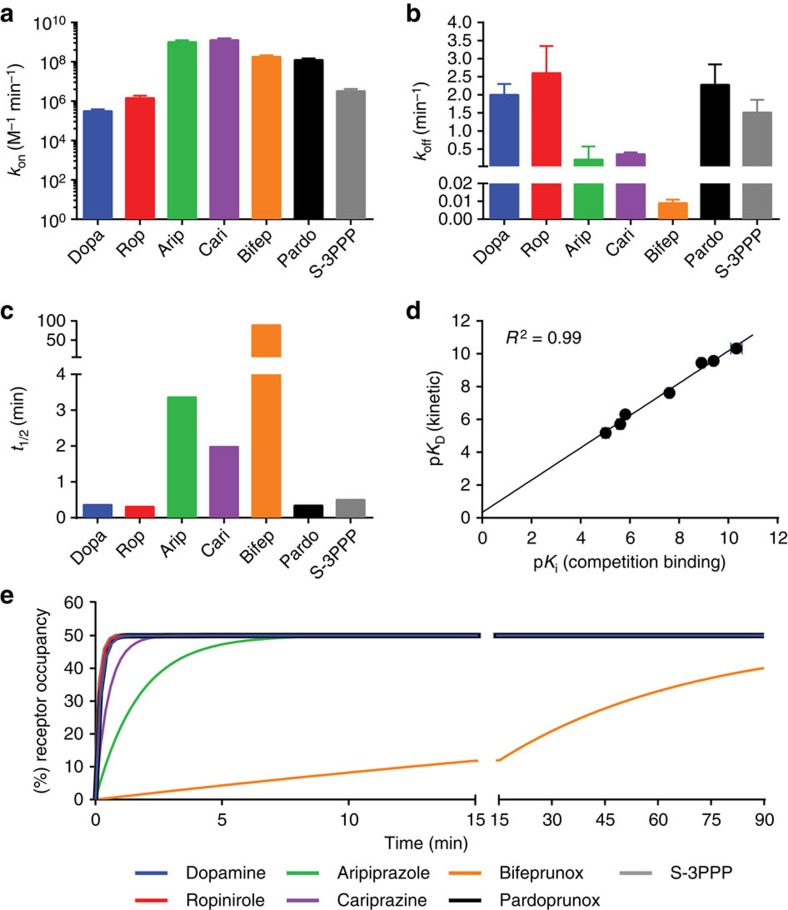
Biased agonists have a longer residence time at the D_2_R compared to dopamine. Kinetic binding parameters of *k*_on_ (**a**), *k*_off_ (**b**) and *t*_1/2_ (**c**) were determined in a competition kinetic binding assay using a Tag-lite binding assay and the fluorescently labelled agonist PPHT as the tracer ligand (Supplementary Table 6). (**d**) A value of affinity for each ligand was calculated using these parameters and compared with that obtained in competition-binding studies using [^3^H]spiperone as the tracer ligand (Supplementary Table 5). All values are expressed as mean±s.e.m. from three experiments. (**e**) The values of *k*_on_ and *k*_off_ obtained in the competition kinetic assay were used to simulate receptor occupancy over time using equation (9) and the concentration of ligand equivalent to its *K*_D_ (see Supplementary Table 6).

We extended these studies with a Tag-lite receptor binding assay using a D_2_R construct with an amino-terminal SNAP tag and the fluorescently labelled agonist (±)-4′-amino-2-(N-phenethyl-N-propyl)-amino-5-hydroxytetralin (PPHT). This ligand displays a faster rate of dissociation (0.45 min^−1^) than that of [^3^H]spiperone and enabled us to obtain accurate values for all ligands (Fig. 4 and Supplementary Table 6). These assays were performed using cell membranes in the presence of 100 μM Gpp(NH)p, to ensure we were measuring binding to the same G protein-uncoupled receptor state as measured using [^3^H]spiperone as a probe. As expected, dopamine, ropinirole, pardoprunox and S-3PPP displayed faster dissociation kinetics than aripiprazole, bifeprunox or cariprazine (Fig. 4b). The association rate of a ligand is often thought to be diffusion limited^34^. However, our data reveal significant variability in the association rate of our selected ligands, with differences of over three orders of magnitude (Fig. 4a and Supplementary Table 6). Therefore, the different affinity of these compounds is driven by both differences in dissociation and association rates^35^. Of note, values of *t*_1/2_ determined for cariprazine, aripiprazole and bifeprunox matched those determined using [^3^H]spiperone as the tracer ligand (Fig. 4c and Supplementary Tables 5 and 6), indicating that the addition of the SNAP tag did not affect the binding of these ligands. Furthermore, values of affinity calculated using these kinetic parameters correlate with values obtained in competition binding experiments with [^3^H]spiperone (Fig. 4d). Using these kinetic parameters, we performed a simulation to estimate the receptor occupancy of a *K*_D_ concentration of each agonist over time (Fig. 4e). This revealed that dopamine, ropinirole, pardoprunox and S-3PPP reached maximal receptor occupancy within 1 min, whereas maximal occupancy for cariprazine and aripiprazole was reached after ∼10 min, and that bifeprunox would not reach maximal occupancy even after 90 min.

### Biased agonism can change profoundly with time

**Figure 5:**
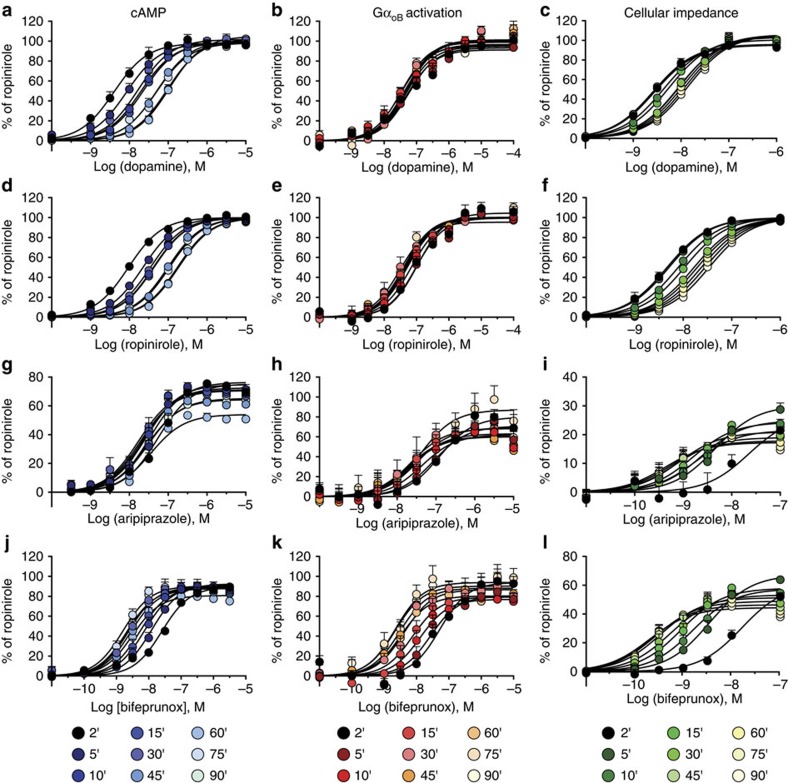
D_2_R agonists display distinct assay-dependent changes in potency over time. The response induced by various concentrations of dopamine (**a**–**c**), ropinirole (**d**–**f**), aripiprazole (**g**–**i**) and bifeprunox (**j**–**l**) by Flp-In-CHO cells stably expressing the D_2L_R was determined at a range of time points between 2 and 90 min. Agonist effects were measured on the inhibition of forskolin-induced cAMP production (**a**,**d**,**g**,**j**), activation of Gα_oB_ G proteins (**b**,**e**,**h**,**k**) and changes in CI (**c**,**f**,**i**,**l**). The data were normalized to the maximal response induced by ropinirole. The values are expressed as mean±s.e.m. from three experiments performed in duplicate; duplicates were averaged before calculating s.e.m. The average potency (pEC_50_) and *E*_max_ for each drug at each time point are displayed in Supplementary Table 7.

It has been previously demonstrated that different signalling pathways have distinct kinetic profiles, for example, the fast or sustained pERK1/2 signal mediated by the β_2_ adrenergic or angiotensin II receptors relating to a G protein versus β arrestin signalling pathway, respectively^36^,^37^. We hypothesized that the different binding kinetics and the temporal pattern of different signalling processes would together manifest in a change in the bias observed for a ligand over time. To address this hypothesis, we took advantage of both the BRET-based biosensors and xCELLigence technology that allow kinetic resolution of intracellular signalling events. We measured the effect of increasing concentrations of agonists on the inhibition of cAMP production, the activation of Gα_oB_ G proteins and CI at various time points ranging from 2 to 90 min (Fig. 5 and Supplementary Fig. 3). Importantly, all agonists maintained a robust signal up to 90 min at these signalling endpoints (Fig. 1).

Ligands with slower dissociation kinetics (aripiprazole and bifeprunox) demonstrated an increase in potency over time in all three assays, which can be attributed to an increase in receptor occupancy over time. Indeed, the order of the three ligands in terms of magnitude of increase in potency over time was the same as the order of their decreasing rate of dissociation (Supplementary Tables 6 and 7). In contrast, we observed a decrease in potency over time for ligands with fast dissociation kinetics (dopamine, ropinirole, S-3PPP and pardoprunox) in both the cAMP and xCELLigence assay, but either no change in potency (dopamine and ropinirole) or a modest increase in potency (pardoprunox: sixfold and S-3PPP: twofold) in the Gα_oB_ activation assay (Supplementary Table 7). This effect cannot be attributed solely to different binding kinetics and suggests that different signalling endpoints are subject to desensitization or regulatory processes that differentially affect the potency and/or efficacy of different ligands over time.

**Figure 6:**
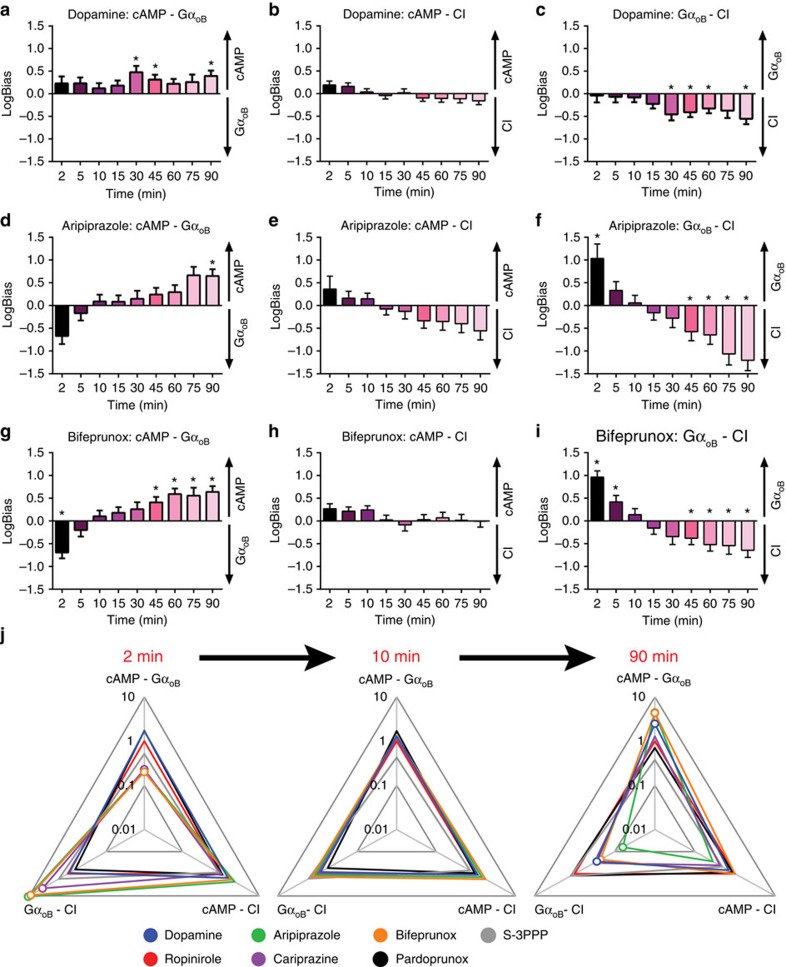
The observed bias determined for aripiprazole changes over time. The concentration–response curves for bifeprunox and aripiprazole at different incubation time points were analysed using an operational model of agonism to obtain transduction coefficients (Log(*τ*/*K*_A_)). These were normalized to the corresponding value obtained for the reference agonist ropinirole at the various time points (ΔLog(*τ*/*K*_A_)) and then the normalized values obtained for one agonist at two different pathways were subtracted to obtain LogBias values (ΔΔLog(*τ*/*K*_A_)) at the various different time points. These LogBias values obtained for dopamine (**a**–**c**), aripiprazole (**d**–**f**) and bifeprunox (**g**–**i**) in reference to ropinirole are represented in bar graphs. Comparisons of agonist action are made between the inhibition of forskolin-stimulated cAMP production versus activation of Gα_oB_ G proteins (**a**,**d**,**g**) or CI (CI, **b**,**e**,**h**), and activation of Gα_oB_ G proteins versus CI (**c**,**f**,**i**). *Significant differences between values of ΔLog(*τ*/*K*_A_) determined at the two different pathways for a particular ligand, determined by a one-way analysis of variance (ANOVA) with a Tukey's post test (*P*<0.05). The changes in overall bias profiles of all compounds are illustrated by webs of bias constructed from data in **a**–**i** at 2, 10 and 90 min. (**j**) The values are expressed as mean±s.e.m. obtained from three experiments performed in duplicate; duplicates were averaged before calculating s.e.m. Open circles indicate significant differences between values of ΔLog(*τ*/*K*_A_) determined at different pathways for a particular ligand, determined by a one-way ANOVA with a Tukey's post test (*P*<0.05).

We used these data to quantify bias at different time points (using ropinirole as the reference agonist), to determine whether these changes in potency over time led to changes in calculated values of bias (Fig. 6 and Supplementary Fig. 4). Although the cognate agonist, dopamine, is released and acts transiently *in vivo*, many synthetic agonists, such as ropinirole, are administered chronically to patients and will therefore produce prolonged D_2_R activation. Furthermore, we could not exclude the possibility that dopamine may be oxidized after long incubation times (ascorbic acid could not be used in our BRET assays due to the interference with luciferase activity). Therefore, for these experiments, we chose to use ropinirole rather than dopamine as the reference agonist. Notably, in the case of bifeprunox and aripiprazole, the direction of bias between pathways was reversed over time (Fig. 6). Bifeprunox displayed significant bias towards the activation of Gα_oB_ G proteins versus inhibition of cAMP production at earlier time points, whereas at later time points it displayed significant bias towards inhibition of cAMP production. Similarly, bifeprunox and aripiprazole were significantly biased towards the activation of Gα_oB_ G proteins versus the CI response at earlier time points, whereas this was reversed at later time points, with a striking 200-fold change in bias for aripiprazole between 2 and 90 min. This change in observed bias must be manifested both by the increase in potency over time observed for the ligands with slower dissociation kinetics (bifeprunox, aripiprazole and cariprazine) and different patterns of changing potency (decreased in cAMP and CI, no change in Gα_oB_ G protein activation) of the reference ligand ropinirole and the other ligands with faster kinetics. These effects also confer changes in the overall pattern of biased agonism between the three pathways, as illustrated by webs of bias generated from measurements taken at 2, 10 and 90-min time points (Fig. 6j). At 2 min, there is a clear distinction between cariprazine, aripiprazole and bifeprunox in comparison with the other ligands driven by their bias towards Gα_oB_ G protein activation in comparison with CI and cAMP. At 10 min, none of the ligands are significantly biased relative to ropinirole. At 90 min, bifeprunox and aripiprazole again show a distinct observed bias profile to ropinirole but with a reversal in direction away from Gα_oB_ G protein activation. Thus, although the direction of observed bias has changed, these ligands retain a bias profile different from that of ropinirole. In contrast, cariprazine displays no bias at 90 min. Interestingly, at this time point, dopamine displays significant bias compared with ropinirole and has a similar bias profile to cariprazine, bifeprunox and aripiprazole, illustrating that even the endogenous agonist has a natural signalling pleiotropy that can change over time.

We hypothesized that both differential ligand binding and signalling kinetics may confer apparent biased agonism if measurements of agonist action at a single pathway are compared at different time points. To explore this, we compared the effect of the agonists at a single pathway (the inhibition of forskolin-stimulated cAMP), whereby agonist effect was measured at a single 30-min time point using an Alphascreen assay (Supplementary Fig. 5), or determined at multiple time points using the BRET-based cAMP biosensor. In agreement with their slow dissociation rate, cariprazine, bifeprunox and aripiprazole all displayed significant ‘bias' towards the measurements of agonist action using the Alphascreen assay measured at 30 min compared with measurements using the BRET biosensor between 2 and 10 min. When measurements were made at a 30-min time point for both assays, all the ligands displayed less than threefold difference in bias compared with ropinirole. Although both cariprazine and aripiprazole did not display significant bias after 30 min, bifeprunox became significantly ‘biased' towards the BRET-based cAMP assay, because the potency of bifeprunox was underestimated at a 30-min time point, in agreement with its much slower rate of receptor occupancy (Fig. 4e).

The time point chosen at which to measure agonist action at a certain pathway often reflects an optimal signal window for a reference agonist. Indeed, the agonist response may be transient in some signalling pathways, as is the case for ERK1/2 phosphorylation where a peak response is observed between 5 and 10 min (Fig. 1). In this case, although the maximal response gained from a ligand with fast dissociation rates such as dopamine will represent high receptor occupancy, this will not be true for slower ligands such as bifeprunox. We quantified biased agonism between ERK1/2 phosphorylation after 5 min of agonist stimulation and the inhibition of forskolin-induced cAMP production after different incubation times (Supplementary Fig. 6). As expected, the ligands with slower dissociation kinetics, aripiprazole, cariprazine and bifeprunox, became more biased towards cAMP inhibition, because they displayed a time-dependent increase in potency for inhibition of cAMP production as opposed to the loss of potency over time observed for ligands such as ropinirole.

### Discrepancies in the biased action of aripiprazole

**Figure 7:**
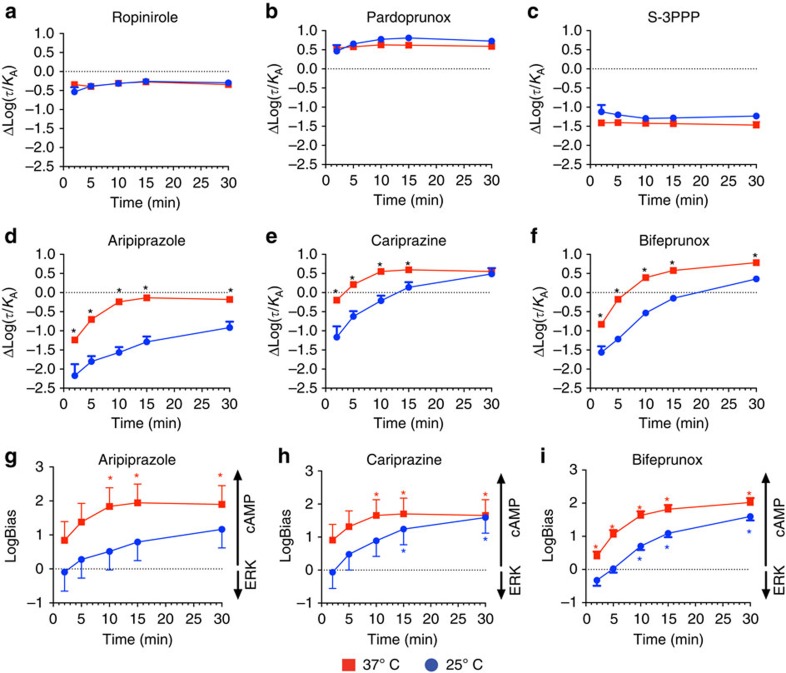
Differences in observation bias can explain discrepancies in prior studies of biased agonism. Experiments measuring ERK phosphorylation using an Alphascreen assay were measured after 5 min of agonist stimulation at 37 °C, whereas experiments measuring inhibition of forskolin-stimulated cAMP using the BRET CAMYEL biosensor were measured at various time points between 2 and 30 min at either 25 or 37 °C. These data were used to determine transduction coefficients (Log(*τ*/*K*_A_)) for the different agonists at the different time points and different assays. The effect of temperature on the ΔLog(*τ*/*K*_A_) values of ropinirole (**a**), pardoprunox (**b**), S-3PPP (**c**), aripiprazole (**d**), cariprazine (**e**) and bifeprunox (**f**) was determined at various time points using the BRET cAMP assay in reference to dopamine. *P<0.05, significant difference between the ΔLog(*τ*/*K*_A_) values determined at 25 and 37 °C as evaluated by a Student's unpaired two-tailed *t*-test. The effect of temperature on biased agonism of aripiprazole (**g**), cariprazine (**h**) and bifeprunox (**i**) over time between ERK1/2 phosphorylation (5 min, 37 °C) and the inhibition of cAMP production (*x*') in reference to dopamine was evaluated. The values are expressed as mean±s.e.m. obtained from three experiments performed in duplicate; duplicates were averaged before calculating s.e.m. *P<0.05, significantly different from the reference agonist dopamine at each time point determined by a Student's unpaired two-tailed *t*-test.

We hypothesized that observational bias caused by differences in experimental conditions may explain prior discrepancies in the action of aripiprazole between two studies, specifically between a recent publication by Szabo *et al*.^17^ and that of Tschammer *et al*.^18^. The former found that aripiprazole was biased towards inhibition of cAMP production versus ERK1/2 phosphorylation as compared with dopamine but the latter did not. Two notable differences in experimental conditions between the studies are the temperature and time at which the cAMP assay was conducted and measured (37 °C and 30 min compared with 25 °C and 15 min, respectively). In contrast, the conditions of the pERK1/2 assay were the same in both studies (5 min agonist stimulation at 37 °C)^17^,^18^. To test this hypothesis, we measured inhibition of forskolin-stimulated cAMP at both 25 and 37 °C. Ligands with similarly fast dissociation kinetics as dopamine (ropinirole, pardoprunox and S-3PPP) were affected in a similar manner by the change in temperature, which resulted in no change in transduction coefficient (Δlog(*τ*/*K*_A_)) values normalized to dopamine (Fig. 7a–c). However, ligands that have slower binding kinetics (aripiprazole, cariprazine and bifeprunox) compared with dopamine, displayed different Δlog(*τ*/*K*_A_) values at 25 °C versus 37 °C (Fig. 7d–f). We then calculated values of LogBias between pERK1/2 (measured after 5 min of agonist stimulation at 37 °C) and inhibition of cAMP production measured at time points between 0 and 30 min. After 15 min at 25 °C (conditions used by Tschammer *et al*.^18^) aripiprazole was not significantly biased towards the inhibition of cAMP production as compared with pERK1/2 (Fig. 7g)^18^. However, after 30 min at 37 °C (conditions used by Szabo *et al*.^17^), aripiprazole displayed significant bias towards inhibition of cAMP^17^. Therefore, we could recapitulate the results of the two studies, demonstrating that both temperature and measurement time underlie the discrepancy in their findings.

## Discussion

To our knowledge, this is the first study to demonstrate that kinetic context, manifested by both ligand-binding kinetics and the kinetics intrinsic to different cellular signalling processes, can profoundly influence observations of biased agonism. This finding has significant implications for the design and interpretation of studies of biased agonism across the drug discovery field^2^,^13^.

Biochemical, biophysical and X-ray crystallography studies have provided evidence of ligand-dependent conformational states of GPCRs^38^,^39^,^40^,^41^,^42^. However, for the majority of studies, a conformation-driven mechanism of biased agonism is inferred indirectly from pharmacological data. This assumption is fundamental to drug-discovery efforts focused on biased agonism^2^,^10^,^27^,^43^. Although evidence of biased agonism has been presented for an ever-increasing number of GPCRs, these data are often inconsistent across different studies^14^,^15^,^16^,^17^,^18^,^44^,^45^. Such discrepancies are indicative of the effect of both observational and system bias on the results of such studies that confound observations of biased agonism. This hampers drug-discovery efforts, because it challenges our ability to correlate biased agonism with drug structure as part of structure–activity studies or, indeed, the physiological effect of a drug.

In the current study, we gave particular focus to the influence of kinetic context on biased agonism. Aripiprazole, cariprazine and bifeprunox displayed slow dissociation kinetics that, in functional assays, conferred an increase in potency over time mediated by an increase in receptor occupancy^29^. In contrast, for the agonists with fast dissociation kinetics, a different pattern of decreasing potency was observed in both the cAMP and CI readouts but not in measurements of Gα_oB_ activation. These different signalling endpoints must thus be subject to pathway- and drug-specific regulatory processes. It is interesting to note that no such decrease in potency was observed for the slow dissociating agonists. Hence, the changes in potency and efficacy over time observed for all of the ligands result from interplay between the kinetics of ligand-binding and cell signalling processes. These changes in potency and efficacy conferred changes in bias over time and, for the slow dissociating ligands aripiprazole and bifeprunox, even a reversal in the direction of bias.

Analytical methods, such as those based on the Black and Leff operational model of agonism, have proved useful to identify biased agonists and allow structure–activity studies to be supplemented with a quantitative measure of bias^9^,^10^,^11^. However, such models assume equilibrium of all reactants. In reality, this is not the case for the majority of functional assays or in physiological systems^3^,^29^. It has been suggested that these methods can eliminate both system and observational bias through the normalization of transduction coefficients to a reference agonist^9^,^10^. The data presented in this study reveal that this approach cannot always eliminate observational bias conferred by differential ligand binding and/or signalling kinetics. The importance of observational bias was further illustrated by the fact that we were able to attribute a discrepancy in the observed biased action of aripiprazole between the study of Szabo *et al*.^17^ and that of Tschammer *et al*.^18^ to differences in experimental conditions and, in particular, temperature and measurement time. It should be noted that the relative contributions of enthalpy and entropy to the binding of an agonist dictate the effect of assay temperature on this binding event^46^. Furthermore, cell signalling processes are also likely to be affected by changes in temperature.

In the majority of studies, the ability of a ligand to elicit a response is measured at a single time point selected based on a maximal signal window obtained for a reference ligand. Such a snapshot is likely to give an incomplete picture of drug action^47^. Indeed, the idea that the observed response of a drug will vary with time has long been acknowledged^20^. Although the majority of signal readouts in our study maintained a robust signal for all agonists over 90 min, ERK1/2 phosphorylation was remarkably more transient in nature for all ligands. Therefore, at the time of maximal signal window, the level of receptor occupancy for slow dissociating agonists such as bifeprunox will be low. Indeed, a recent study at the serotonin 5-HT_2B_ receptor demonstrated that the potency of slow dissociating agonists was underestimated when transient signalling events were measured^48^. Thus, agonists with slow dissociation kinetics may display an apparent bias towards pathways that are measured at longer time, because receptor occupancy will be higher. We observe such a pattern for the slow dissociating agonists when comparing their action in a pERK1/2 assay measured at 5 min and a cAMP assay measured at longer time points.

A number of studies have attempted to relate measurements of biased agonism to ligand structure, to build structure–activity relationships of biased agonism^15^,^43^,^49^,^50^,^51^,^52^. The majority of such studies are performed in heterologous systems with the expectation that a bias phenotype (for example, bias towards a β-arrestin pathway over a G protein-dependent pathway) will be maintained in the (patho)physiologically relevant cell background or tissue. Our finding that the magnitude and direction of bias may change over time adds a further layer of complexity to the interpretation of such studies. In this study, although drugs (bifeprunox, cariprazine and aripiprazole) that displayed a distinct bias profile at 5 min remained biased relative to ropinirole at 90 min, the direction of bias changed. In such cases, ascribing directionality to the description of a biased ligand (for example, bias towards G protein activation) may be an insufficient descriptor of the action of a drug, as bias determined at an acute time point may differ considerably from the bias observed after longer drug administration. Indeed, descriptions of bias direction should be qualified with the conditions in which this bias was detected including time point(s).

**Figure 8:**
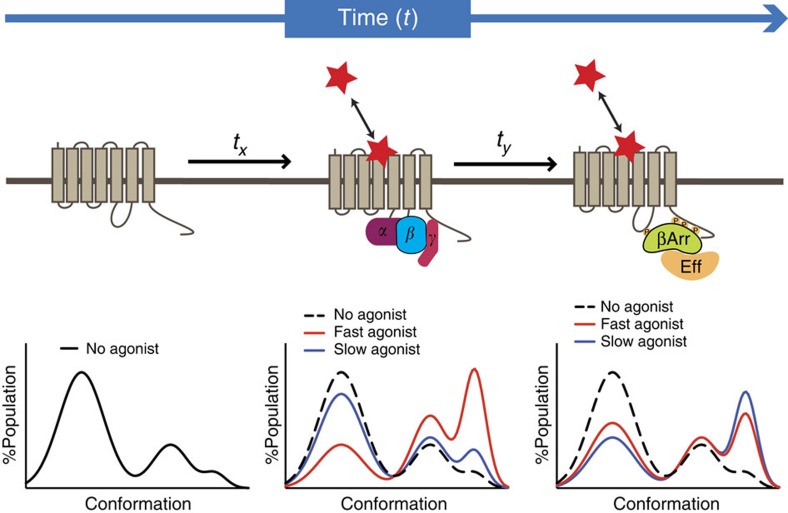
The impact of kinetic bias on the energy landscape of a GPCR. A receptor may adopt different conformations as it engages different signalling effector and regulatory proteins. Agonists may have different association and dissociation kinetics, which determine the residence time of the agonist on the receptor. The duration of a ligand–receptor complex may determine the different effector and regulatory proteins that can be engaged over time and thus the conformational landscape that can be explored by that agonist–receptor complex over time.

Our data do not exclude a mechanism by which biased agonism is conferred by the stabilization of distinct receptor conformations by different ligands. Such a mechanism may be concomitant with differential binding kinetics, whereby the duration of a ligand–receptor complex determines the different effector and regulatory proteins that can be engaged over time. Thus, we propose ‘kinetic bias' as a mechanism that can modify the apparent energy landscape of GPCRs at different time points (Fig. 8). Indeed, an endogenous ligand may have a ‘natural' signalling pleiotropy that changes over time as the agonist–receptor complex engages distinct transducer proteins, that is, G proteins or β arrestins. Other agonists acting at the same receptor may engage signalling and regulatory pathways to different extents in a time-dependent manner. Although conformational selection (by both ligand and transducer) must be the fundamental underlying mechanism by which biased agonism is manifested, our findings suggest that ligand-binding kinetics relative to signalling endpoints must now be routinely considered. A number of studies have demonstrated the ability of some receptors to maintain sustained signalling following internalization^47^,^53^,^54^,^55^. Of interest, recent studies have suggested a link between long ligand residence time and both the prolonged internalization of the S1P_1_ receptor and persistent signalling from internalized 5-HT_2B_ receptors and calcitonin receptors^48^,^56^,^57^. It is interesting to note that the three ligands that displayed slower dissociation kinetics than dopamine have all shown antipsychotic efficacy in clinical trials, and that slow dissociation kinetics of D_2_R partial agonists have been linked to reduced prolactin release^58^. Although the propensity of antagonist antipsychotic drugs to cause extrapyramidal side effects has been linked to their dissociation rate from the D_2_R, future studies are required to understand the link between the dissociation rate of D_2_R partial agonists and their therapeutic efficacy.

Our findings suggest that the linking of a cellular mechanism of biased agonism to the physiological effect of a drug represents a significant challenge. Ideally, one could screen for biased ligands in the physiologically relevant tissue and provide unequivocal evidence that is the biased action of a ligand that leads to its differential action. However, such an approach is not practical for the screening of a large number of compounds and requires a detailed understanding of the physiological system and, in particular, the relationship between different signalling pathways and physiological effect. In the vast majority of cases, such information is incomplete or missing. Thus, the screening of ligands at multiple signalling endpoints in a simple cell system that allows one to study the action of drugs at a single GPCR target, in combination with analytical methods (such as those based on the operational model of agonism) to quantify this agonist action and to allow comparison across different pathways, represents a practical compromise. These measurements can then be used to ‘cluster' ligands based on their effects across different pathways. Based on our findings, we now propose that selected ligands from each cluster be subsequently assessed in terms of kinetic profiling, to identify those that may be expressing their bias predominantly through a kinetic mechanism. Finally, exemplar molecules can then be progressed into more complex, physiologically relevant systems to see whether this bias profile confers differential effects in this system. Therefore, we propose that an operational approach to quantify drug action together with the measurement of ligand-binding kinetics may allow a more informed clustering of compounds. This in turn will provide an improved foundation for drug discovery of biased agonists.

## Methods

### Materials

Aripiprazole and cariprazine were synthesized in-house as previously described^7^,^52^. Bifeprunox and pardoprunox were synthesized in-house as described in the Supplementary Methods. All compounds were shown to be >95% pure. Ropinirole was purchased from BetaPharma (Shanghai) Co. Ltd (Wujiang, China) and >98% pure as described by the supplier. S-3PPP and dopamine were purchased from Sigma-Aldrich (Castle Hill, NSW, Australia) and are >98% pure as indicated by the supplier. The G protein BRET constructs were generated by Dr Céline Galés (Paul Sabatier University, France)^59^. The pcDNA3L-His-CAMYEL was purchased from ATCC. The Rluc8-tagged D_2L_R and YFP–β-arrestin-2 constructs were a gift from Associate Professor Michelle Glass (University of Auckland, New Zealand). DMEM medium (DMEM) and Flp-In-CHO cells were purchased from Invitrogen (Carlsbad, CA). Fetal bovine serum was purchased from ThermoTrace (Melbourne, VIC, Australia). [^3^H]Spiperone, AlphaScreen reagents and Ultima gold scintillation cocktail were purchased from PerkinElmer (Boston, MA). The Tag-lite labelling medium (LABMED), SNAP-Lumi4-Tb and the PPHT derivative labelled with a red fluorescent probe (PPHT-red) was from Cisbio Bioassays (Bagnols-sur-Cèze, France). Three hundred and eighty-four-well Optiplates plates were purchased from PerkinElmer (Beaconsfield, UK). Spiperone and DMEM/Nutrient F-12 Ham) were obtained from Sigma Chemical Co Ltd. (Poole, UK); all other cell culture reagents including pluronic acid, Hank's-based cell dissociation buffer and PBS were obtained from GIBCO (Invitrogen, Paisley, UK). All of the other reagents were purchased from Sigma-Aldrich (Castle Hill, NSW, Australia).

### Cell lines and transfection

Flp-In-CHO cells were grown in DMEM supplemented with 10% fetal bovine serum and maintained at 37 °C in a humidified incubator containing 5% CO_2_. The Flp-In-CHO cells were transfected with the pOG44 vector encoding Flp recombinase and the pDEST vector encoding the D_2L_R at a ratio of 9:1 using polyethylenimine as the transfection reagent^60^. Twenty-four hours after transfection, the cells were subcultured and the medium was supplemented with 700 μg ml^−1^ HygroGold (Invivogen) as selection agent, to obtain cells stably expressing the D_2L_R.

### Bioluminescence resonance energy transfer

*β-Arrestin-2 recruitment*. Flp-In-CHO cells were seeded at a density of 2,000,000 cells per 10-cm dish and were transfected the following day using polyethylenimine as the transfection reagent. To assess β-arrestin-2 recruitment to the D_2L_R, the cells were transfected with 1 μg Rluc8-tagged D_2L_R, 4 μg YFP–β-arrestin-2 and 2 μg GRK2. Twenty-four hours after transfection, the cells were plated into 96-well CulturPlates (PerkinElmer) and grown overnight. The cells were equilibrated in Hank's balanced salt solution at 37 °C before starting the experiment. The cells were incubated with Coelenterazine (Promega) at a final concentration of 5 μM for 5 min, followed by stimulation with the agonists for an additional 5 min before the BRET readings were captured. The signals were detected at 445–505 and 505–565 nm using a LUMIstar Omega instrument (BMG LabTech, Offenburg, Germany). Net BRET was determined by subtraction of the vehicle control from the agonist-induced response.

*Cyclic AMP*. Flp-In-CHO cells stably expressing the D_2L_R were seeded at a density of 2,000,000 cells per 10-cm dish and were transfected the following day using polyethylenimine as the transfection reagent. The cells were transfected with 3 μg CAMYEL, to allow the detection of cAMP levels within the cells. Twenty-four hours after transfection, the cells were plated into 96-well CulturPlates (PerkinElmer) and grown overnight. The cells were equilibrated in Hank's balanced salt solution at 37 °C (or 25 °C when indicated) before starting the experiment. The cells were co-stimulated with the agonists and 10 μM forskolin for the indicated timeframes when the BRET readings were captured. Coelenterazine (Promega) was added at a final concentration of 5 μM at least 3 min before measurement. The signals were detected at 445–505 and 505–565 nm using a LUMIstar Omega instrument (BMG LabTech). Net BRET was determined by subtraction of the vehicle control co-added with 10 μM forskolin.

*G protein activation*. Flp-In-CHO cells stably expressing the D_2L_R were seeded at a density of 2,000,000 cells per 10-cm dish and were transfected the following day using polyethylenimine as the transfection reagent. To measure the activation of Gα_i1_ and Gα_OB_ G proteins, the cells were transfected with either 0.3 μg Rluc8-tagged Gα_i1_, 1.2 μg Gβ and 1.35 μg Venus-tagged Gγ, or 0.14 μg Rluc8-tagged Gα_OB_, 1.2 μg Gβ and 0.6 μg Venus-tagged Gγ. Twenty-four hours after transfection, the cells were plated into 96-well CulturPlates (PerkinElmer) and grown overnight. The cells were equilibrated in Hank's balanced salt solution at 37 °C before starting the experiment. The cells were stimulated with the agonists for the indicated timeframes when the BRET readings were captured. Coelenterazine (Promega) was added at a final concentration of 5 μM at least 3 min before measurement. The signals were detected at 445–505 and 505–565 nm using a PHERAstar FS instrument (BMG LabTech). Net BRET was determined by subtraction of the vehicle control from the agonist-induced response.

### xCELLigence (CI) assay

Changes in CI on ligand stimulation were measured using the xCELLigence system (Roche Applied Science). The baseline cell index of each well of a 96-well E-plate was measured in the presence of growth medium before cell seeding. Cells were seeded at a density of 20,000 cells per well and grown for 16–20 h followed by 4 h serum starvation. Cells were treated with the ligands and the cell index values were obtained immediately after ligand stimulation every 15 s for a total time of 90 min. The retrieved cell index values were then normalized by dividing the cell index at the time of ligand addition followed by subtraction of the vehicle control at each time point.

### ERK1/2 phosphorylation assay

ERK1/2 phosphorylation was measured using the Alphascreen SureFire ERK kit (PerkinElmer, Waltham, USA). Cells were seeded into 96-well plates at a density of 50,000 cells per well. After 5–7 h, cells were washed with PBS and incubated in serum-free DMEM overnight before assaying. Dose–response experiments were performed for each ligand at 37 °C in the presence of 0.1% ascorbic acid. Stimulation of the cells was terminated after 5 min of agonist stimulation by removing the media and the addition of 100 μl of SureFire lysis buffer to each well. The plate was shaken for 5 min at room temperature (RT) before transferring 5 μl of the lysates to a white 384-well Proxiplate (PerkinElmer, Waltham, USA). Next, 8 μl of a 240:1,440:7:7 mixture of Surefire activation buffer:Surefire reaction buffer:Alphascreen acceptor beads:Alphascreen donor beads was added to the samples and incubated in the dark at 37 °C for 1.5 h. Plates were read using a Fusion-TM plate reader (PerkinElmer, Waltham, USA).

### cAMP Alphascreen assay

Flp-In-CHO cells stably expressing the D_2L_R were grown overnight in 96-well plates at a density of 50,000 cells per well. After pre-incubating the cells for 45 min with stimulation buffer (Hank's buffered salt solution: 0.14 M NaCl, 5.4 mM KCl, 0.8 μM MgSO_4_, 1.3 mM CaCl_2_, 0.2 mM Na_2_HPO_4_, 0.44 mM KH_2_PO_4_, 5.6 mM D-glucose, 1 mg ml^−1^ BSA, 0.5 mM 3-isobutyl-1-methylxanthine and 5 mM HEPES pH 7.4), the cells were stimulated simultaneously with drug and 300 nM forskolin for 30 min at 37 °C. Stimulation of cells was terminated by the removal of the stimulation buffer and the addition of 50 μl ice-cold 100% ethanol. The plates containing the cell lysates were then incubated at 37 °C without lid, to allow complete evaporation of the ethanol. After all the ethanol was evaporated, 50 μl of detection buffer (1 mg ml^−1^ BSA, 0.3% Tween-20 and 5 mM HEPES pH 7.4) was added to each well. The plate was shaken for 5 min, to ensure complete and even suspension of the cell material. Five microlitres of the sample was then transferred into a white 384-well Optiplate (PerkinElmer, Waltham, USA). Anti-cAMP acceptor beads (0.2 units per μl) diluted in stimulation buffer were added to all samples and incubated in the dark at RT for 30 min before addition of 15 μl of the donor beads/biotinylated cAMP (0.07 units per μl) mixture made up in detection buffer. Following a 1-h incubation at RT, plates were read using a Fusion-TM plate reader (PerkinElmer, Waltham, USA).

### Membrane preparation

Flp-In-CHO cells stably expressing the dopamine D_2L_R were grown to 90% confluency in 175 cm^2^ cell culture flasks. The cells were harvested in PBS containing 2 mM EDTA and centrifuged at 300 *g* for 3 min. The resulting pellet was resuspended in ice-cold assay buffer (20 mM HEPES, 100 mM NaCl, 6 mM MgCl_2_, 1 mM EGTA and 1 mM EDTA pH 7.4) and the centrifugation step was repeated. The intact cell pellet was then resuspended in assay buffer and homogenized using a Polytron homogenizer. After centrifugation (1,000 *g*, 10 min), the pellet was discarded and the supernatant was recentrifuged at 30,000 *g* for 1 h at 4 °C using a Sorvall Evolution RC ultracentrifuge (Thermo Scientific). The resulting pellet was resuspended in assay buffer and stored in 250 μl aliquots at −80 °C. Membrane protein concentration was determined using the method of Bradford.

### [^3^H]Spiperone-binding assays

All radioligand binding experiments were conducted at 37 °C in a 1-ml reaction volume in assay buffer (20 mM HEPES, 100 mM NaCl, 6 mM MgCl_2_, 1 mM EGTA and 1 mM EDTA pH 7.4) containing 100 μM GppNHp and 0.1% ascorbic acid. In all cases, nonspecific binding was determined in the presence of 10 μM haloperidol. After the indicated incubation period, bound and free [^3^H]spiperone were separated by fast-flow filtration through GF/B filters using a brandel harvester followed by three washes with ice-cold 0.9% NaCl. Filter-bound radioactivity was measured by scintillation spectrometry after the addition of 3.5 ml of Ultima Gold (PerkinElmer) using a Tri-Carb 2900TR liquid scintillation counter (PerkinElmer).

The dissociation rate of [^3^H]spiperone was determined by allowing 0.2 nM [^3^H]spiperone to reach equilibrium with Flp-In-CHO-D_2L_ membranes (10 μg) in a final volume of 1 ml after which re-association of [^3^H]spiperone was prevented by the addition of haloperidol. Bound [^3^H]spiperone was measured at multiple time points after the addition of haloperidol. The *k*_off_ determined for [^3^H]spiperone was 0.026 min^−1^.

Association binding experiments were conducted to determine the *k*_obs_ of [^3^H]spiperone. Association was initiated by the addition of 0.2 nM [^3^H]spiperone to Flp-In-CHO-D_2L_R membranes (10 μg) and terminated by filtration. The *k*_on_ of [^3^H]spiperone was derived from the *k*_obs_ using the *k*_off_ value predetermined in dissociation binding experiments. The *k*_on_ determined for [^3^H]spiperone was 9.51 × 10^8^ M^−1^ min^−1^.

The kinetic parameters of unlabelled agonists were determined using the equations described by Motulsky and Mahan^31^. Both [^3^H]spiperone and unlabelled agonist were simultaneously added to Flp-In-CHO-D_2L_R membranes (10 μg). The amount of [^3^H]spiperone bound to the receptor was assessed at multiple time points. To ensure that the rate parameters calculated for the unlabelled agonists were independent of ligand concentration, the experiment was performed in the presence of two or three different concentrations of unlabelled agonist.

To obtain affinity estimates of unlabelled agonists, [^3^H]spiperone competition experiments were performed at equilibrium. The ability of increasing concentrations of the agonists to compete with 0.1 nM [^3^H]spiperone for binding to the D_2L_R was tested. The membranes (Flp-In-CHO-D_2L_R, 5 μg) were incubated with the drugs for 3 h at 37 °C. The *K*_i_ values of the unlabelled agonists were determined using the *K*_D_ of [^3^H]spiperone (0.028 nM), which was obtained from kinetic experiments.

### Cell culture and terbium labelling of SNAP-tagged D_2L_ cells

Chinese hamster ovary cells transfected with the complementary DNA encoding a SNAP-tagged human dopamine D_2L_ (SNAP-CHO-D_2L_) receptor were maintained in DMEM/Nutrient F-12 Ham, 2 mM glutamine supplemented 10% FCS. To terbium label the SNAP-CHO-D_2L_ cells, cell culture medium was removed from the T175 cm^2^ flasks containing confluent adherent SNAP-CHO-D_2L_ cells. Twelve millilitres of Tag-lite labelling medium containing 100 nM of SNAP-Lumi4-Tb was added to the flask and incubated for 1 h at 37 °C under 5% CO_2_. Cells were then washed 2 × in 15 ml PBS (GIBCO, Invitrogen, Paisley, UK) to remove the excess of SNAP-Lumi4-Tb and detached using 5 ml of enzyme-free Hank's-based cell dissociation buffer (GIBCO, Invitrogen, Paisley, UK) and collected in a vial containing 5 ml of DMEM/Nutrient F-12 Ham, 2 mM glutamine supplemented 10% FCS. Cells were pelleted by centrifugation (5 min at 1,500 r.p.m.) and the pellets were frozen to −80 °C before preparation of membranes.

### Membrane preparation of terbium-labelled cells

All subsequent steps described were conducted at 4 °C, to avoid receptor degradation. Twenty millilitres of wash buffer (10 mM HEPES and 10 mM EDTA pH 7.4) was added to the pellet of terbium-labelled cells (equivalent to 1 × *t*175 cm^2^). This was homogenized using an electrical homogenizer Ultra-Turrax (Ika-Werk GmbH & Co. KG, Staufen, Germany) (position 6, 5 × 1 s bursts) and subsequently centrifuged at 48,000 *g* at 4 °C (Beckman Avanti J-251 Ultracentrifuge; Beckman Coulter, Fullerton, CA) for 30 min. The supernatant was discarded and the pellet was homogenized and centrifuged as described above in wash buffer. The final pellet was suspended in ice-cold 10 mM HEPES and 0.1 mM EDTA pH 7.4, at a concentration of 5–10 mg m^−1^. Protein concentration was determined by the bicinchoninic acid assay using BSA as a standard and aliquots maintained at −80 °C until required. Before their use, the frozen membranes were thawed and the membranes suspended in the assay buffer at a membrane concentration of 0.1 mg m^−1^.

### Fluorescent ligand-binding assays

All fluorescent binding experiments using PPHT-red were conducted at 37 °C in 384-well Optiplates plates, in assay binding buffer, 20 mM HEPES, 100 mM NaCl, 6 mM MgCl_2_, 1 mM EGTA, 1 mM EDTA and pluronic acid 0.02% pH 7.4, containing 100 μM GppNHp and 0.1% ascorbic acid. GppNHp was included to remove the G protein-coupled population of receptors that can result in two binding sites in membrane preparations, because the Motulsky–Mahan model is only appropriate for ligands competing at a single site. In all cases, nonspecific binding was determined in the presence of 1 μM spiperone. Both fluorescent ligands and unlabelled compounds were diluted in the binding assay buffer.

Time resolved fluorescence resonance energy transfer signals were acquired in a PHERAstarFS plate reader (BMG Labtech) equipped with a Homogeneous Time Resolved Fluorescence module capable of excitation at 337 nm and emission at 620 and 665 nm. Kinetics acquisitions at intervals of 20 s were recorded using the standard settings of the reader for HTRF measurements. The Terbium donor was excited with three laser flashes at a wavelength of 337 nm and time-resolved recording of the acceptor and donor emission took place at 620 and 665 nm, respectively, using the default settings of the instrument. HTRF ratios were obtained by dividing the acceptor signal (665 nm) by the donor signal (620 nm) and multiplying this value by 10,000. Specific binding was determined by subtracting the nonspecific HTRF ratio from the total HTRF ratio.

### Determination of PPHT-red binding kinetics

To accurately determine association rate (*k*_on_) and dissociation rate (*k*_off_) values, the observed rate of association (*k*_ob_) was calculated using at least four different concentrations of PPHT-red. The appropriate concentration of PPHT-red was incubated in a 384-well Optiplate with human SNAP-D_2L_R-CHO cell membranes (2 μg per well) in assay binding buffer at 37 °C (final assay volume, 40 μl). The degree of PPHT-red bound to the receptor was assessed at multiple time points by HTRF detection, to allow construction of association kinetic curves. The resulting data were globally fitted to the association kinetic model (equation (8)) to derive a single best-fit estimate for *k*_on_ and *k*_off_ as described under ‘Data analysis'.

### Competition binding kinetics

On the same 384-well Optiplate plate, the kinetic parameters of unlabelled ligand were assessed using a competition kinetic binding assay. This approach involves the simultaneous addition of both fluorescent ligand and competitor to receptor preparation so that at *t*=0 all receptors are unoccupied. PPHT-red (12.5 nM; a concentration that avoids ligand depletion in this assay volume) was added simultaneously with the unlabelled compound (at *t*=0) to CHO cell membranes containing the human D_2L_ (2 μg per well) in 40 μl of assay buffer. The degree of PPHT-red bound to the receptor was assessed at multiple time points by HTRF detection. Nonspecific binding was determined as the amount of HTRF signal detected in the presence of spiperone (1 μM) and was subtracted from each time point. Each time point was conducted on the same 384-well plate incubated at 37 °C with shaking (100 r.p.m.) after every cycle.

Multiple concentrations of unlabelled competitor were tested, for determination of rate parameters based up on the affinities determined from the [^3^H]spiperone competition binding assays described above. Data were globally fitted using equation (7), to simultaneously calculate *k*_on_ and *k*_off_. Different ligand concentration ranges were chosen, as compounds with a long residence time equilibrate more slowly; thus, a higher relative concentration is required to ensure the experiments reach equilibrium within a reasonable time frame, while still maintaining a good signal-to-noise. *K*_i_ values of the compounds were determined from competitive binding experiments according to the Cheng and Prusoff equation^61^ as described under ‘Data analysis'.

### Data analysis

The results were analysed using Prism 6.0 (GraphPad Software Inc., San Diego, CA).

Dose–response curves were fitted using the following three parameter equation


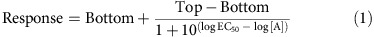


where Top and Bottom represent the maximal and minimal asymptote of the dose–response curve, [A] is the molar concentration of agonist and EC_50_ is the molar concentration of agonist required to give a response half way between bottom and top. Dose–response data were fitted to the following form of the operational model of agonism^12^ to allow the quantification of biased agonism


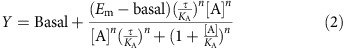


where *E*_m_ is the maximal possible response of the system, Basal is the basal level of response, *K*_A_ represents the equilibrium dissociation constant of the agonist (A) and *τ* is an index of the signalling efficacy of the agonist that is defined as *R*_T_/*K*_E_, where *R*_T_ is the total number of receptors and *K*_E_ is the coupling efficiency of each agonist-occupied receptor, and *n* is the slope of the transducer function that links occupancy to response. The analysis assumes that the transduction machinery used for a given cellular pathway are the same for all agonists, such that the *E*_m_ and transducer slope (*n*) are shared between agonists. Data for all compounds for each pathway were fit globally, to determine values of *K*_A_ and *τ.* Biased agonism was quantified as previously described^62^. In short, to exclude the impact of cell-dependent and assay-dependent effects on the observed agonism at each pathway, the log(*τ*/*K*_A_) value of a reference agonist, in this case ropinirole or dopamine, is subtracted from the log(*τ*/*K*_A_) value of the agonists of interest to yield Δlog(*τ*/*K*_A_). The relative bias can then be calculated for each agonist at the two different signalling pathways by subtracting the Δlog(*τ*/*K*_A_) of one pathway from the other to give a ΔΔlog(*τ*/*K*_A_) value, which is a measure of bias. A lack of biased agonism will result in values of ΔΔlog(*τ*/*K*_A_) not significantly different from 0 between pathways. All affinity (p*K*_i_ or p*K*_A_), potency (pEC_50_) and transduction ratio (log(*τ*/*K*_A_)) parameters were estimated as logarithms. When fold changes in bias are described, this was calculated by converting values of ΔΔlog(*τ*/*K*_A_) to the corresponding antilog value. However, we have previously demonstrated that such a distribution of these parameters does not conform to a normal (Gaussian) distribution, whereas the logarithm of the measure is approximately Gaussian^63^. Thus, as the application of *t*-tests and analyses of variance assume Gaussian distribution, estimating the parameters as logarithms allows valid statistical comparison. All results are expressed as the mean±s.e.m. As such, we performed a Brown–Forsythe test (Graphpad prism 6) to assure ourselves of equal variance when such parameters are compared.

The concentration of agonist that inhibited half of the [^3^H]spiperone binding (IC_50_) was determined using the following equation


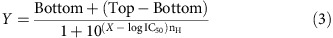


where *Y* denotes the percentage-specific binding, Top and Bottom denote the maximal and minimal asymptotes, respectively, IC_50_ denotes the *X*-value when the response is midway between Bottom and Top, and *n*_H_ denotes the Hill slope factor. IC_50_ values obtained from the inhibition curves were converted to *K*_i_ values using the Cheng and Prusoff equation^61^.

The dissociation data were fitted to a one-phase exponential decay function and the *t*_1/2_ value obtained was transformed into a *k*_off_ rate using the following equation:


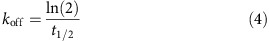


[^3^H]spiperone association data were fitted to a single-phase exponential association function to calculate an observed rate constant *k*_ob_. The association rate constant, *k*_on_, was calculated as described originally by Hill^63^:


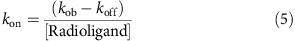


where the *k*_off_ value used was predetermined from dissociation experiments. These parameters can then be used to calculate the radioligand equilibrium dissociation constant, *K*_D_:


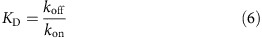


Association and dissociation rates for unlabelled agonists were calculated by fitting the equations described by Motulsky and Mahan^31^:


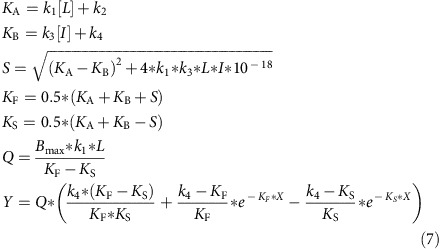


where *X* is time (min), *Y* is specific binding, *k*_1_ is *k*_on_ [^3^H]spiperone (M^−1^ min^−1^), *k*_2_ is *k*_off_ [^3^H]spiperone (min^−1^), *L* is the concentration of [^3^H]spiperone used (M) and *I* is the concentration of unlabelled agonist (M). Fixing the above parameters allowed the calculation of the following parameters: the maximum number of receptor sites *B*_max_, the association rate of the unlabelled agonist *k*_3_ (M^−1^ min^−1^) and the dissociation rate of the unlabelled agonist *k*_4_ (min^−1^).

PPHT-red association data were globally fitted to equation (8), where *L* is the concentration of ligand in nM using GraphPad Prism 6.0, to determine a best-fit estimate for *k*_on_ and *k*_off_.


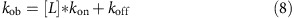


Association and dissociation rates for unlabelled agonists were calculated by fitting to equation (7), described above for [^3^H]-spiperone binding, except *k*_1_ is *k*_on_ PPHT-red (M^−1^ min^−1^), *k*_2_ is *k*_off_ PPHT-red (min^−1^), *L* is the concentration of PPHT-red used (M).

The simulation displayed in Fig. 3 was performed using the equation


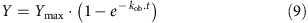


where *Y*_max_ reflects maximum binding of a concentration of ligand and *k*_ob_ is determined by equation (8).

### Principal component analysis

PCA is a dimensionality reduction method that uses transformations to project a high-dimensional set of data into a lower dimensional set of variables called principal components. The principal components extract the important information from the data, revealing its internal structure in a way that best explains its variance^28^. The PCA was applied using singular value decomposition as implemented in the package scikit-learn^64^.

## Additional information

**How to cite this article:** Klein Herenbrink, C. *et al*. The role of kinetic context in apparent biased agonism at GPCRs. *Nat. Commun.* 7:10842 doi: 10.1038/ncomms10842 (2016).

## Supplementary Material

Supplementary Information Supplementary Figures 1-6, Supplementary Tables 1-7, Supplementary Methods and Supplementary References

Supplementary Data 1 Excel file of raw data relating to the time course experiments shown in Figure 1.
