# Supplementary material for: The role of kinetic context in apparent biased agonism at GPCRs
Source: Nat Commun. 2016 Feb 24;7:10842. doi: 10.1038/ncomms10842 (PMC4770093; doi:10.1038/ncomms10842)
Supplement: Supplementary Information — Supplementary Figures 1-6, Supplementary Tables 1-7, Supplementary Methods and Supplementary References [file ncomms10842-s1.pdf]

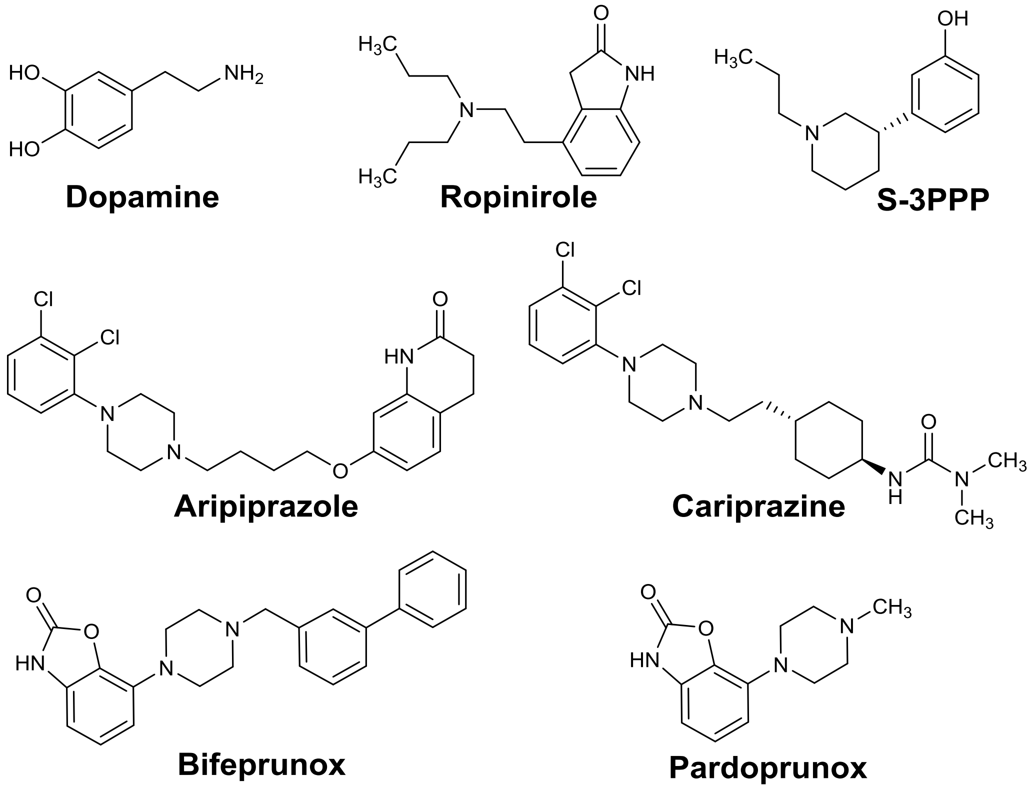

Supplementary Figure 1. Chemical structures of the reference agonists, dopamine and ropinirole, and the partial agonists S-3PPP, aripiprazole, cariprazine, bifeprunox and pardoprunox.

.

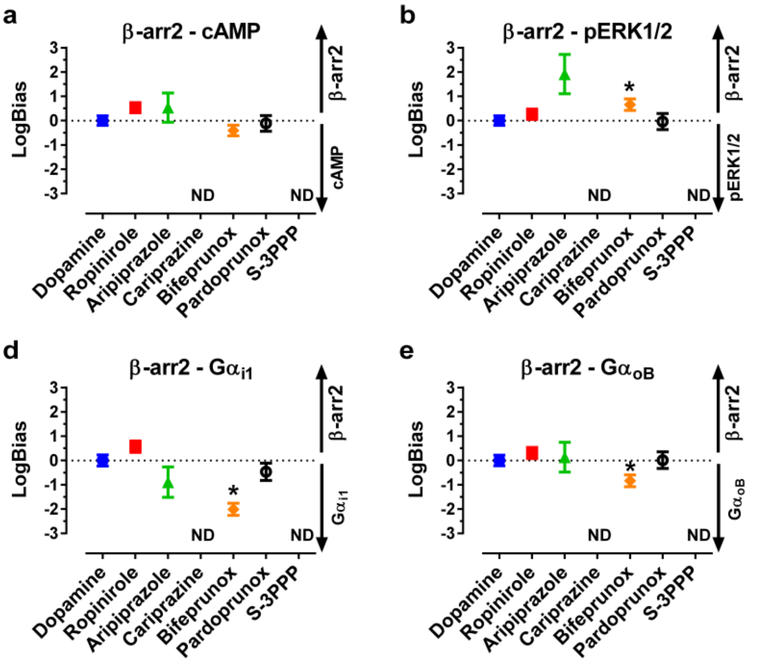

Supplementary Figure 2. Quantification of biased agonism at the D_2R between the recruitment of β-arrestin-2 and various other signalling pathways at 5 minutes. LogBias values were determined between β-arrestin-2 (β-arr2) recruitment and the inhibition of forskolin-induced cAMP production (**a**), ERK1/2 phosphorylation (**b**), induced changes in cellular impedance (CI) (**c**), and the activation of $G\alpha_{i1}$ (**d**) and $G\alpha_{oB}$ (**e**) G proteins for various agonists. The values are represented in supplementary table 4 and are expressed as mean ± S.E.M. from three experiments performed in duplicate, duplicates were averaged before calculating S.E.M.. ND, no LogBias values were determined for cariprazine and S-3PPP due to the lack of a measurable agonist response in the β-arrestin-2 recruitment assay. *$P<0.05$, significantly different from the reference agonist dopamine determined by a Student's unpaired two-tailed t-test.

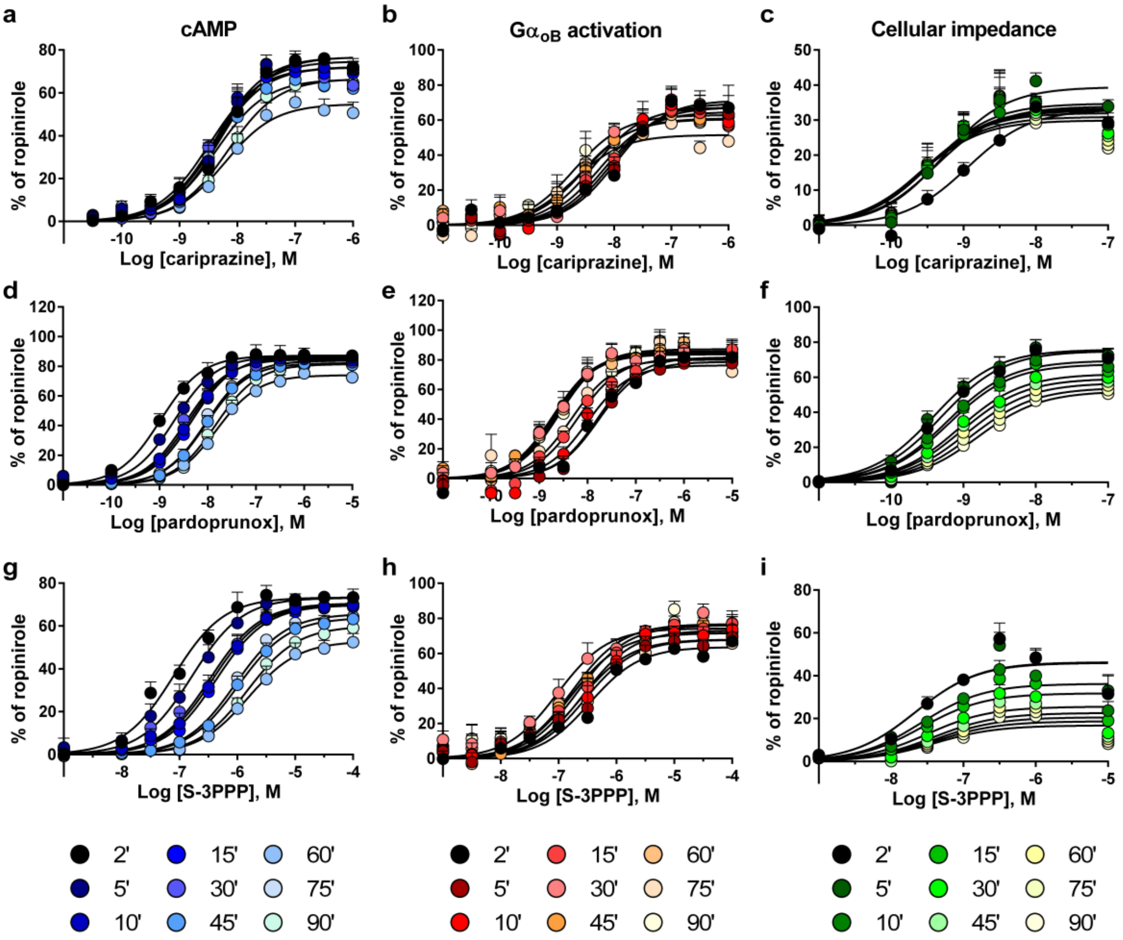

Supplementary Figure 3. The D_2R agonists dopamine, cariprazine, pardoprunox and S-3PPP display distinct assay-dependent changes in potency over time. The response induced by various concentrations of cariprazine (**a-c**), pardoprunox (**d-f**) and S-3PPP (**g-i**) by Flp-In-CHO cells stably expressing the $D_{2L}R$ was determined at a range of timepoints between 2 and 90 minutes. Agonist effects were measured upon the inhibition of forskolin-induced cAMP production (**a, d, g**), activation of Gα_{oB} (**b, e, h**) and changes in cellular impedance (**c, f, i**). The data was normalized to the maximal response induced by ropinirole. The values are expressed as mean ± S.E.M. from three experiments performed in duplicate, duplicates were averaged before calculating S.E.M..

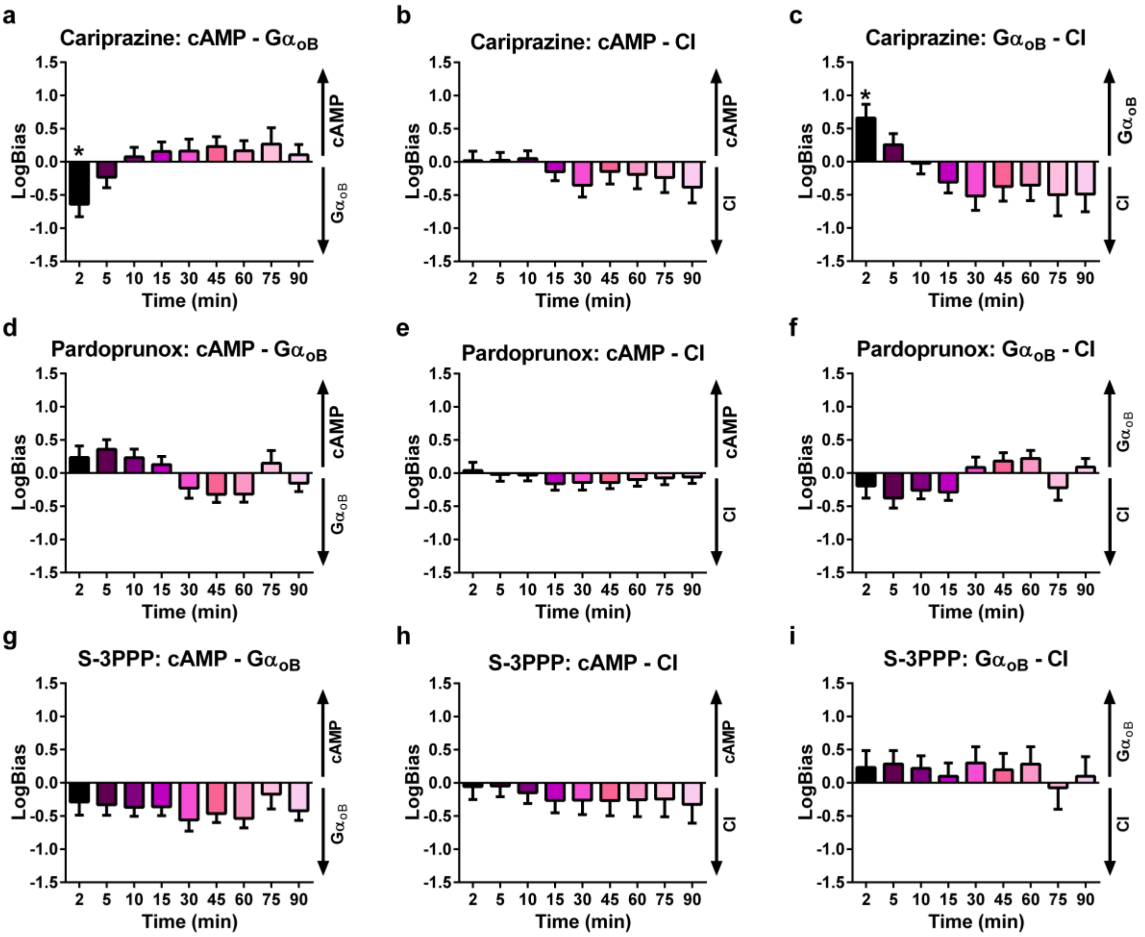

Supplementary Figure 4. The observed profile of biased agonism determined for cariprazine changes over time whereas no change is observed for the fast dissociating ligands pardoprunox and S-3PPP. The concentration-response curves for cariprazine, pardoprunox and S-3PPP at different incubation timepoints (Supplementary Figure 5) were analysed using an operational model of agonism to obtain transduction coefficients ($Log(\tau/K_A)$). These were normalized to the corresponding value obtained for the reference agonist ropinirole at the various timepoints ($\Delta Log(\tau/K_A)$) and then the normalized values obtained for one agonist at two different pathways were subtracted to obtain LogBias values ($\Delta\Delta Log(\tau/K_A)$) at the various different timepoints. These LogBias values obtained for cariprazine (**a-c**), pardoprunox (**d-f**) and S-3PPP (**g-i**) in reference to ropinirole are represented in bar graphs. Comparisons of agonist action are made between the inhibition of forskolin-stimulated cAMP production versus activation of $G\alpha_{oB}$ G proteins (**a, d & g**) or

cellular impedance (CI, **b, e & h**) and activation of $G\alpha_{oB}$ G proteins versus cellular impedance (**c, f & i**). The values are expressed as mean ± S.E.M. obtained from three experiments performed in duplicate, duplicates were averaged before calculating S.E.M.. *$P<0.05$, significantly different from the reference agonist ropinirole determined by a one-way ANOVA with Tukey's post test.

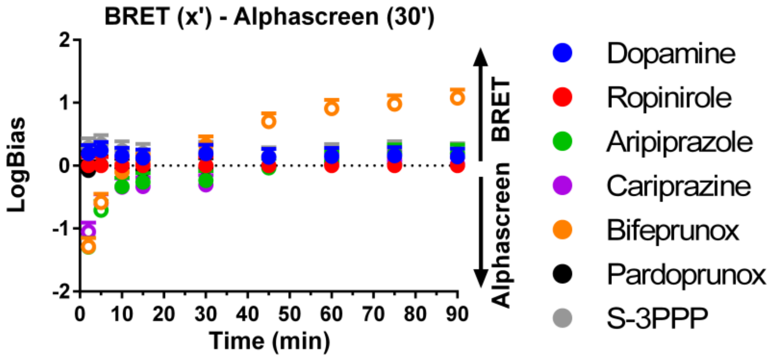

Supplementary Figure 5. Time-dependent changes in the observed profiles of apparent biased agonism between two assays measuring inhibition of forskolin-stimulated cAMP production. Comparison of the BRET cAMP assay using the CAMYEL biosensor (x') and the Alphascreen^{TM} cAMP assay (30') indicate that the slower associating ligands bifeprunox, aripiprazole and cariprazine display significant apparent bias towards the Alphascreen^{TM} assay up to 30 minutes while at the 30 minute time-point in the CAMYEL BRET assay all ligands tested had a similar efficacy (< 3-fold difference). Only bifeprunox, which had the slowest association kinetics, became significantly more biased towards the cAMP BRET assay after 30 minutes. The values are expressed as mean ± S.E.M. obtained from three experiments performed in duplicate, duplicates were averaged before calculating S.E.M.. Open circles indicate significant differences from the reference agonist ropinirole determined by a Student's unpaired two-tailed t-test (P<0.05).

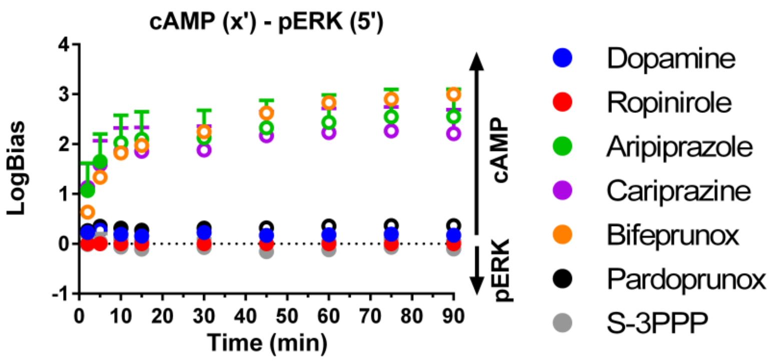

Supplementary Figure 6. Comparison of the action of dopaminergic ligands between ERK phosphorylation measured at 5 minutes and the inhibition of forskolin-induced cAMP production over time. The effect of incubation time in the BRET cAMP assay on calculated values of LogBias for various dopaminergic ligands between the inhibition of cAMP production measured at various timepoints between 2 and 90 minutes after agonist addition (x') and ERK1/2 phosphorylation measured at 5 minutes (5') in reference to ropinirole was determined. The values are expressed as mean ± S.E.M. obtained from three experiments performed in duplicate, duplicates were averaged before calculating S.E.M.. Open circles indicate significant differences from the reference agonist ropinirole determined by a Student's unpaired two-tailed t-test ($P < 0.05$).

Supplementary table 1. Log(*τ*/K_A) values determined for D_2R agonists at different signalling pathways

	Dopamine	Ropinirole	Aripiprazole	Cariprazine	Bifeprunox	Pardoprunox	S-3PPP
cAMP	8.06 ± 0.05	7.67 ± 0.04	7.36 ± 0.06	8.27 ± 0.06	7.88 ± 0.05	8.64 ± 0.05	6.65 ± 0.06
pERK1/2	8.56 ± 0.05	8.44 ± 0.05	6.48 ± 0.54	7.45 ± 0.47	7.32 ± 0.10	9.06 ± 0.08	7.39 ± 0.14
$G\alpha_{i1}$ activation	7.17 ± 0.09	6.75 ± 0.09	7.90 ± 0.16	8.10 ± 0.14	8.60 ± 0.11	8.10 ± 0.13	6.60 ± 0.12
$G\alpha_{oB}$ activation	7.24 ± 0.08	7.08 ± 0.08	6.94 ± 0.11	7.91 ± 0.11	7.50 ± 0.10	7.70 ± 0.10	6.40 ± 0.11
Cellular impedance	8.55 ± 0.05	8.32 ± 0.05	7.83 ± 0.15	8.88 ± 0.11	8.30 ± 0.07	9.29 ± 0.06	7.34 ± 0.10
β-arrestin2	6.33 ± 0.13	6.48 ± 0.15	6.17 ± 0.58	ND	5.75 ± 0.16	6.80 ± 0.29	ND

Log(*τ*/K_A) values were determined of D_2R agonists for the inhibition of forskolin-induced cAMP production, induced changes in cellular impedance, ERK1/2 phosphorylation (pERK1/2), $G\alpha_{i1}$ and $G\alpha_{oB}$ G protein activation and β-arrestin2 recruitment from experiments performed at a 5 minute timepoint. Values are expressed as mean ± S.E.M. of three experiments performed in duplicate, duplicates were averaged before calculating S.E.M.. ND = no agonist activity detected in the β-arrestin-2 recruitment assay.

Supplementary table 2. $\Delta Log(\tau/K_A)$ values determined for D_2R agonists at different signalling pathways using dopamine as reference agonist

	Dopamine	Ropinirole	Aripiprazole	Cariprazine	Bifeprunox	Pardoprunox	S-3PPP
cAMP	0.00 ± 0.06	-0.39 ± 0.06	-0.70 ± 0.07	0.21 ± 0.07	-0.18 ± 0.07	0.58 ± 0.07	-1.40 ± 0.08
pERK1/2	0.00 ± 0.07	-0.12 ± 0.07	-2.08 ± 0.55	-1.10 ± 0.47	-1.24 ± 0.11	0.50 ± 0.10	-1.17 ± 0.15
$G\alpha_{i1}$ activation	0.00 ± 0.13	-0.43 ± 0.13	0.73 ± 0.18	0.93 ± 0.17	1.43 ± 0.14	0.93 ± 0.16	-0.58 ± 0.15
$G\alpha_{oB}$ activation	0.00 ± 0.11	-0.16 ± 0.11	-0.30 ± 0.14	0.67 ± 0.14	0.26 ± 0.13	0.45 ± 0.13	-0.84 ± 0.14
Cellular impedance	0.00 ± 0.07	-0.23 ± 0.06	-0.72 ± 0.16	0.33 ± 0.12	-0.25 ± 0.08	0.74 ± 0.07	-1.22 ± 0.11
β-arrestin2	0.00 ± 0.19	0.14 ± 0.20	-0.17 ± 0.60	ND	-0.58 ± 0.21	0.47 ± 0.32	ND

$\Delta Log(\tau/K_A)$ values were determined of D_2R agonists using dopamine as reference agonist for the inhibition of forskolin-induced cAMP production, induced changes in cellular impedance, ERK1/2 phosphorylation (pERK1/2), $G\alpha_{i1}$ and $G\alpha_{oB}$ G protein activation and β-arrestin2 recruitment from experiments performed at a 5 minute timepoint. Values are expressed as mean ± S.E.M. of three experiments performed in duplicate, duplicates were averaged before calculating S.E.M.. ND = no agonist activity detected in the β-arrestin-2 recruitment assay.

Supplementary Table 3. Quantification of biased agonism at the D_2R using the operational model of agonism

	Dopamine	Ropinirole	Aripiprazole	Cariprazine	Bifeprunox	Pardoprunox	S-3PPP
cAMP – CI	0.00 ± 0.09	-0.16 ± 0.09	0.02 ± 0.18	-0.12 ± 0.14	0.08 ± 0.11	-0.16 ± 0.10	-0.19 ± 0.13
$G\alpha_{oB}$ – cAMP	0.00 ± 0.13	0.23 ± 0.13	0.40 ± 0.16	0.46 ± 0.16	0.43 ± 0.14	-0.13 ± 0.15	0.56 ± 0.16
cAMP – pERK1/2	0.00 ± 0.10	-0.27 ± 0.10	1.38 ± 0.55*	1.32 ± 0.48*	1.06 ± 0.13*	0.08 ± 0.12	-0.24 ± 0.17
$G\alpha_{i1}$ – cAMP	0.00 ± 0.15	-0.03 ± 0.15	1.43 ± 0.20*	0.71 ± 0.19	1.61 ± 0.16*	0.35 ± 0.17	0.83 ± 0.17*
$G\alpha_{oB}$ – CI	0.00 ± 0.13	0.07 ± 0.13	0.42 ± 0.21	0.35 ± 0.19	0.51 ± 0.15*	-0.29 ± 0.15	0.37 ± 0.18
CI – pERK1/2	0.00 ± 0.10	-0.11 ± 0.10	1.36 ± 0.57*	1.43 ± 0.49*	0.99 ± 0.14*	0.24 ± 0.12	-0.05 ± 0.18
$G\alpha_{i1}$ – CI	0.00 ± 0.15	-0.19 ± 0.15	1.45 ± 0.25*	0.60 ± 0.21	1.68 ± 0.16*	0.19 ± 0.18	0.64 ± 0.19*
$G\alpha_{oB}$ – pERK1/2	0.00 ± 0.14	-0.04 ± 0.14	1.78 ± 0.56*	1.78 ± 0.49*	1.50 ± 0.17*	-0.05 ± 0.16	0.33 ± 0.20
$G\alpha_{i1}$ – $G\alpha_{oB}$	0.00 ± 0.18	-0.26 ± 0.18	1.03 ± 0.23	0.25 ± 0.22	1.17 ± 0.19*	0.48 ± 0.21	0.27 ± 0.21
$G\alpha_{i1}$ – pERK1/2	0.00 ± 0.15	-0.31 ± 0.15	2.81 ± 0.58*	2.03 ± 0.50*	2.67 ± 0.18*	0.43 ± 0.19	0.59 ± 0.21*

LogBias values were determined for D_2R agonists between the inhibition of cAMP production (cAMP), changes in cellular impedance (CI), ERK1/2 phosphorylation (pERK1/2), $G\alpha_{i1}$ activation and $G\alpha_{oB}$ G protein activation using dopamine as reference agonist from experiments performed in all assays at a 5 minute timepoint. Values are expressed as mean ± S.E.M. of three experiments performed in duplicate, duplicates were averaged before calculating S.E.M.. *P < 0.05, * indicates significant differences between values of $\Delta Log(\tau/K_A)$ determined at the two different pathways for a particular ligand, determined by a one-way ANOVA with a Tukey's post test (P < 0.05).

Supplementary Table 4. Quantification of biased agonism between β-arrestin2 recruitment (βarr2) and other signalling pathways at the D_2R using the operational model of agonism

	Dopamine	Ropinirole	Aripiprazole	Cariprazine	Bifeprunox	Pardoprunox	S-3PPP
β-arr2 – cAMP	0.00 ± 0.20	0.54 ± 0.21	0.54 ± 0.60	ND	-0.41 ± 0.22	-0.12 ± 0.33	ND
β-arr2 – pERK1/2	0.00 ± 0.20	0.26 ± 0.21	1.92 ± 0.81	ND	0.66 ± 0.24	-0.04 ± 0.33	ND
β-arr2 – CI	0.00 ± 0.20	0.38 ± 0.21	0.56 ± 0.62	ND	-0.33 ± 0.22	-0.28 ± 0.33	ND
β-arr2 – $G\alpha_{i1}$	0.00 ± 0.23	0.57 ± 0.24	-0.89 ± 0.62	ND	-2.01 ± 0.25	-0.47 ± 0.36	ND
β-arr2 – $G\alpha_{oB}$	0.00 ± 0.22	0.31 ± 0.23	0.14 ± 0.61	ND	-0.84 ± 0.24	0.01 ± 0.34	ND

LogBias values were determined for D_2R agonists between the inhibition of cAMP production (cAMP), changes in cellular impedance (CI), ERK1/2 phosphorylation (pERK1/2), $G\alpha_{i1}$ and $G\alpha_{oB}$ G protein activation and β-arrestin2 recruitment (βarr2) using dopamine as reference agonist from experiments performed in all assays at a 5 minute timepoint. Values are expressed as mean ± S.E.M. of three experiments performed in duplicate, duplicates were averaged before calculating S.E.M..*P < 0.05, significantly different from the reference agonist dopamine determined by a Student's unpaired two-tailed t-test. ND = no agonist activity detected in the β-arrestin-2 recruitment assay.

Supplementary Table 5. Binding parameters of D_2R agonists derived from competition association binding experiments using [^{3}H]spiperone as the tracer ligand

Ligand	k_{on} (M^{-1} min^{-1})	k_{off} (min^{-1})	pK_d[a]	$t_{1/2}$ (mins)	pK_i[b]
Dopamine	ND	ND	-	ND	5.05 ± 0.06
Ropinirole	ND	ND	-	ND	5.60 ± 0.08
Aripiprazole	1.31 ± 0.15 x 10^8	0.14 ± 0.02	8.97	4.95	9.43 ± 0.06
Cariprazine	1.55 ± 0.42 x 10^7	0.49 ± 0.14	7.50	1.42	8.90 ± 0.07
Bifeprunox	1.07 ± 0.11 x 10^8	0.01 ± 0.00	10.0	86	10.36 ± 0.09
Pardoprunox	ND	ND	-	ND	7.63 ± 0.06
S-3PPP	ND	ND	-	ND	5.84 ± 0.06

k_{on} and k_{off} values were determined with competition association binding experiments. Values are expressed as mean ± S.E.M. from three separate experiments. ND = Accurate values for dopamine, ropinirole, pardoprunox and S-3PPP could not be determined due to the slow dissociation of [^{3}H]spiperone. [a] value of affinity determined from kinetic parameters. [b] value of affinity determined from competition binding experiments using [^{3}H]spiperone.

Supplementary Table 6. Binding parameters of D_2R agonists derived from competition association binding experiments using fluorescent PHTH as the tracer ligand and a Tag-lite^{TM} binding assay

Ligand	k_{on} (M^{-1} min^{-1})	k_{off} (min^{-1})	pK_d [a]	$t_{1/2}$ (mins)
Dopamine	3.14 ± 0.73 x 10^5	2.00 ± 0.30	5.18	0.36
Ropinirole	1.46 ± 0.46 x 10^6	2.60 ± 0.75	5.73	0.31
Aripiprazole	1.01 ± 0.23 x 10^9	0.21 ± 0.02	9.66	3.36
Cariprazine	1.27 ± 0.30 x 10^9	0.35 ± 0.05	9.53	1.98
Bifeprunox	1.84 ± 0.30 x 10^8	0.01 ± 0.00	10.3	89.1
Pardoprunox	1.25 ± 0.24 x 10^8	2.28 ± 0.56	7.75	0.34
S-3PPP	3.25 ± 0.90 x 10^6	1.51 ± 0.35	6.11	0.50

k_{on} and k_{off} values were determined with competition association binding experiments. Values are expressed as mean ± S.E.M. from three separate experiments. [a] value of affinity determined from kinetic parameters.

Supplementary Table 7. The potency and E_{max} (relative to the maximal response of ropinirole) of D_2R agonists in assays measuring inhibition of forskolin stimulated cAMP production (cAMP), activation of Gαo1 G proteins and stimulation of cellular impedence (CI) measured at time points between 2 and 90 minutes

Assay		Ropinirole		Dopamine		Aripiprazole		Cariprazine		Bifeprunox		Pardoprunox		S-3PPP	
		pEC_{50}	E_{max}	pEC_{50}	E_{max}	pEC_{50}	E_{max}	pEC_{50}	E_{max}	pEC_{50}	E_{max}	pEC_{50}	E_{max}	pEC_{50}	E_{max}
**cAMP**	**2’**	8.02 ± 0.02	100	8.40 ± 0.05	98 ± 1.8	7.26 ± 0.06	75 ± 1.9	8.30 ± 0.07	77 ± 2.4	7.60 ± 0.06	89 ± 2.0	8.98 ± 0.07	87 ± 1.9	7.10 ± 0.07	73 ± 1.9
	**5’**	7.65 ± 0.02*	100	8.07 ± 0.06*	98 ± 2.0	7.47 ± 0.07*	76 ± 2.0	8.38 ± 0.07	77 ± 2.4	7.91 ± 0.05*	92 ± 2.0	8.69 ± 0.05*	87 ± 1.5	6.78 ± 0.07	73 ± 2.0
	**10’**	7.38 ± 0.02*	100	7.71 ± 0.04*	98 ± 1.6	7.58 ± 0.05*	75 ± 1.6	8.38 ± 0.07	75 ± 2.4	8.13 ± 0.06*	91 ± 2.0	8.39 ± 0.04*	86 ± 1.4	6.43 ± 0.06*	70 ± 1.7
	**15’**	7.37 ± 0.02*	100	7.65 ± 0.04*	99 ± 1.6	7.65 ± 0.06*	72 ± 1.6	8.38 ± 0.08	72 ± 2.5	8.27 ± 0.07*	89 ± 2.4	8.33 ± 0.05*	87 ± 1.6	6.37 ± 0.06*	70 ± 1.9
	**30’**	7.46 ± 0.02*	100	7.78 ± 0.06*	102 ± 2.0	7.76 ± 0.08*	71 ± 2.3	8.49 ± 0.09	72 ± 2.6	8.62 ± 0.05*	89 ± 1.7	8.46 ± 0.07*	85 ± 2.1	6.47 ± 0.09*	71 ± 2.5
	**45’**	7.01 ± 0.02*	100	7.29 ± 0.04*	101 ± 1.7	7.56 ± 0.07*	64 ± 1.9*	8.37 ± 0.07	66 ± 1.9*	8.56 ± 0.06*	88 ± 2.0	8.03 ± 0.04*	82 ± 1.4	5.99 ± 0.05*	64 ± 1.4*
	**60’**	6.73 ± 0.03*	100	6.97 ± 0.05*	105 ± 2.0	7.45 ± 0.08	54 ± 1.7*	8.22 ± 0.10	55 ± 2.4*	8.51 ± 0.07*	81 ± 2.0	7.82 ± 0.06*	74 ± 1.8*	5.82 ± 0.09*	53 ± 2.4*
	**75’**	6.97 ± 0.03*	100	7.30 ± 0.06*	98 ± 2.0	7.71 ± 0.08*	71 ± 2.1	8.40 ± 0.07	72 ± 2.0	8.80 ± 0.09*	90 ± 2.5	8.04 ± 0.06*	84 ± 2.2	6.04 ± 0.05*	66 ± 1.4
	**90’**	6.76 ± 0.03*	100	7.03 ± 0.04*	102 ± 2.0	7.53 ± 0.07	65 ± 1.8*	8.16 ± 0.06	67 ± 1.8*	8.69 ± 0.08*	88 ± 2.5	7.83 ± 0.05*	81 ± 1.6	5.82 ±0.03 *	60 ± 0.8*
**Ga_o**	**2’**	6.97 ± 0.08	100	7.26 ± 0.10	93 ± 3.6	6.91 ± 0.18	80 ± 6.0	7.97 ± 0.14	71 ± 4.7	7.28 ± 0.09	94 ± 3.8	7.78 ± 0.13	82 ± 4.5	6.46 ± 0.22	64 ± 5.6
	**5’**	7.02 ± 0.06	100	7.31 ± 0.07	91 ± 2.0	7.07 ± 0.17	71 ± 5.0	8.05 ± 0.12	70 ± 3.7	7.57 ± 0.11	80 ± 3.6*	7.77 ± 0.13	79 ± 4.3	6.54 ± 0.11	68 ± 3.0
	**10’**	7.17 ± 0.06	100	7.40 ± 0.06	97 ± 2.0	7.36 ± 0.16	62 ± 4.0	8.14 ± 0.10	67 ± 3.0	7.89 ± 0.10*	77 ± 3.0*	8.00 ± 0.10	76 ± 3.2	6.57 ± 0.11	73 ±3.1

	**15’**	7.33 ± 0.05*	100	7.49 ± 0.06	95 ± 2.0	7.60 ± 0.15	63 ± 4.0	8.25 ± 0.11	65 ± 3.0	8.12 ± 0.10*	79 ± 2.8*	8.21 ± 0.09	81 ± 2.8	6.67 ± 0.07	76 ± 2.2
	**30’**	7.51 ± 0.07*	100	7.30 ± 0.07	101 ± 2.8	7.64 ± 0.20*	68 ± 5.0	8.36 ± 0.14	67 ± 3.0	8.39 ± 0.08*	87 ± 2.5	8.70 ± 0.10*	84 ± 3.1	7.02 ± 0.11*	74 ± 3.0
	**45’**	7.38 ± 0.05*	100	7.35 ± 0.07	100 ± 2.6	7.72 ± 0.16*	60 ± 3.7*	8.55 ± 0.12*	61 ± 3.0	8.52 ± 0.08*	88 ± 2.4	8.71 ± 0.10*	85 ± 3.1	6.78 ± 0.08	72 ± 2.2
	**60’**	7.29 ± 0.05*	100	7.35 ± 0.07	101 ± 2.8	7.68 ± 0.15*	60 ± 3.6*	8.58 ± 0.12*	60 ± 2.8	8.49 ±0.07*	80 ± 1.9*	8.64 ± 0.08*	86 ± 2.7	6.79 ± 0.08	72 ± 2.3
	**75’**	7.40 ± 0.08*	100	7.47 ± 0.10	99 ± 3.8	7.39 ± 0.18	87 ± 6.4	8.71 ± 0.22*	52 ± 4.5	8.66 ± 0.14*	94 ± 4.0	8.34 ± 0.16*	80 ± 4.9	6.62 ± 0.12	68 ±2 .9
	**90’**	7.36 ±* 0.08	100	7.33 ± 0.07	95 ± 2.4	7.49 ± 0.14	69 ± 4.0	8.71 ± 0.13*	63 ± 3.2	8.67 ± 0.11*	90 ± 3.2	8.58 ± 0.08*	87 ± 2.5	6.77 ± 0.09	77 ± 2.6
**CI**	**2’**	8.33 ± 0.04	100	8.55 ± 0.06	95 ± 2.4	7.65 ± 0.35	27 ± 7.0	8.94 ± 0.13	33 ± 2.3	7.79 ± 0.10	61 ± 4	9.30 ± 0.10	75 ± 3.6	7.66 ± 0.19	46 ± 3.7
	**5’**	8.29 ± 0.03	100	8.58 ± 0.05	96 ± 1.9	8.34 ± 0.16	30 ± 3.0	9.28 ± 0.10	39 ± 1.9	8.46 ± 0.07*	67 ± 3	9.41 ± 0.08	76 ± 2.7	7.67 ± 0.16	46 ± 3.0
	**10’**	8.10 ± 0.03*	100	8.36 ± 0.04	101 ± 2.1	8.62 ± 0.12*	25 ± 2.0	9.38 ± 0.11	34 ± 1.7	8.80 ± 0.08*	57 ±2.5	9.22 ± 0.07	70 ± 2.2	7.58 ± 0.16	36 ± 2.5
	**15’**	7.97 ± 0.03*	100	8.25 ± 0.05*	103 ± 2.2	8.80 ± 0.13*	24 ± 1.7	9.45 ± 0.12	35 ± 2.0	9.04 ± 0.09*	58 ± 2.8	9.20 ± 0.07	67 ± 2.5	7.58 ± 0.19	32 ± 2.6*
	**30’**	7.77 ± 0.03*	100	8.05 ± 0.06*	105 ± 3.0	8.74 ± 0.16*	22 ± 2.0	9.50 ± 0.17	33 ± 2.5	9.26 ± 0.12*	53 ± 3.1	9.06 ± 0.09	62 ± 2.9*	7.50 ± 0.22	26 ± 2.5*
	**45’**	7.71 ± 0.04*	100	8.03 ± 0.05*	106 ± 2.9	9.09 ± 0.17*	21 ± 1.8	9.52 ± 0.19	33 ± 2.8	9.47 ± 0.10*	51 ± 2.3	9.02 ± 0.08	59 ± 2.4*	7.41 ± 0.24	23 ± 2.5*
	**60’**	7.62 ± 0.04*	100	7.97 ± 0.06*	106 ± 3.1	9.17 ± 0.19*	19 ± 1.8	9.55 ± 0.22	32 ± 3.0	9.58 ± 0.10*	49 ± 2.2*	8.95 ± 0.07*	57 ± 2.2*	7.39 ± 0.26	21 ± 2.5*
	**75’**	7.55 ± 0.03*	100	7.91 ± 0.06*	106 ± 3.3	9.28 ± 0.20*	18 ± 1.7	9.57 ± 0.23	31 ± 3.2	9.64 ± 0.11*	46 ± 2.2*	8.87 ± 0.07*	54 ± 2.1*	7.40 ± 0.28	19 ± 2.4*
	**90’**	7.46 ± 0.05*	100	7.86 ± 0.06*	106 ± 3.5	9.37 ± 0.21*	17 ± 1.7	9.60 ± 0.25	30 ± 3.2	9.70 ± 0.12*	44 ± 2.*1	8.80 ± 0.07*	52 ± 2.0*	7.41 ± 0.30	17 ± 2.3 *

Values are expressed as mean ± S.E.M. of three experiments performed in duplicate, duplicates were averaged before calculating S.E.M.. * = significant differences relative to the parameter measured at 2 minutes for each ligand in each assay (one way ANOVA with dunnet’s post hoc test, P < 0.05

Supplementary Methods

Chemistry

All reagents were purchased from Sigma-Aldrich, Alfa Aesar, AK Scientific or Chem-Impex International, and used without purification. GR grade ammonium hydroxide solution (28% aqueous solution), and LR grade methanol, ethyl acetate, chloroform, DCM and acetonitrile were purchased from Merck and used without further purification. All ^{1}H NMR and ^{13}C NMR spectra (DEPTQ) were recorded on a Bruker Avance III 400 Ultrashield Plus spectrometer at 400.13 and 100.62 MHz respectively. Results were recorded as follows: chemical shift values are expressed as δ units generally acquired in $CDCl_3$, CD_3OD or d_6-DMSO where specified, with tetramethylsilane (0.00 ppm) as reference for ^{1}H NMR (residual solvent peak as reference for ^{13}C NMR),[1] multiplicity (singlet (s), doublet (d), triplet (t), quartet (q), broad (br), multiplet (m), doublet of doublets (dd), doublet of triplets (dt), triplet of triplets (tt), quartet of doublets (qd)), apparent (app), coupling constants (*J*) in Hertz and integration. Thin-layer chromatography was conducted on 0.2 mm plates using Merck silica gel 60 F_{254}. Flash Chromatography was performed using Merck Silica Gel 60, 230-400 mesh ASTM. High resolution mass spectra (HRMS) were obtained on a Waters LCT Premier XE (TOF) using electrospray ionization (ESI) at a cone voltage of 50 V. LCMS data were obtained on an Agilent 1200 series LC coupled directly to a photodiode array detector and an Agilent 6100 Quadrupole MS, using a Phenomenex® column (Luna 5 µm C8, 50 mm × 4.60 mm ID). Analytical reverse-phase HPLC was performed on a Waters HPLC system coupled directly to a photodiode array detector and fitted with a Phenomenex® Luna C8 (2) 100 Å column (150 mm × 4.6 mm, 5 µm) using a binary solvent system; solvent A: 0.1% TFA/H_2O; solvent B: 0.1% TFA/80% CH_3CN/H_2O. Gradient elution was achieved using 100%

solvent A to 100% solvent B over 20 min at a flow rate of 1 mL/min. All compounds were >95 % purity by HPLC (λ = 254, 214 nm) prior to biological testing.

5-Chloro-7-nitrobenzo[*d*]oxazol-2(3*H*)-one[2]

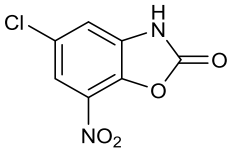

2-Amino-4-chloro-6-nitrophenol (5.00 g, 26.5 mmol) was taken up in EtOAc (60 mL), and 1,1′-carbonyldiimidazole (4.73 g, 29.2 mmol) was added. The solution was heated to 50 °C, and stirred at this temperature for 24 h. The mixture was then cooled, filtered, and the brown precipitate washed with water (3 × 30 mL), conc. HCl (3 × 20 mL), 1 M NaOH (3 × 20 mL), water (3 × 20 mL) then collected and dried *in vacuo* overnight to reveal the title compound as a brown solid (4.26 g, 75%).

^{1}H NMR (400 MHz, d_6-DMSO) δ 7.28 (d, *J* = 2.1 Hz, 1H), 7.07 (d, *J* = 2.1 Hz, 1H). ^{13}C NMR (101 MHz, d_6-DMSO) δ 153.5 (C), 136.8 (C), 134.5 (C), 131.2 (C), 127.6 (C), 116.1 (CH), 115.5 (CH).

7-Aminobenzo[*d*]oxazol-2(3*H*)-one[2]

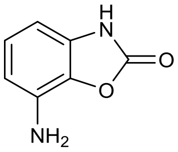

A two-necked flask was evacuated of atmosphere and refilled with nitrogen gas three times. 10% Pd/C (248 mg, 233 μmol) was added under inert atmosphere, then DCM slowly added until the solid was fully submerged, followed by slow addition of absolute EtOH (30 mL). 5-Chloro-7-nitrobenzo[*d*]oxazol-2(3*H*)-one (1.00 g, 4.66 mmol) was then added, and the flask evacuated of atmosphere. A hydrogen balloon was attached, and the evacuation/filling process with hydrogen was repeated three

times and left stirring under an atmosphere of hydrogen at 60 °C for 24 h. After this time, the mixture was filtered through a Celite® 545 pad and washed with absolute EtOH (40 mL). The filtrate was evaporated to dryness to reveal the title compound as a brown solid (588 mg, 84%), which was recrystallised from water to give brown flakes.

^{1}H NMR (400 MHz, d_6-DMSO) δ 11.29 (s, 1H), 6.82 (app t, J = 7.9 Hz, 1H), 6.39 (dd, J = 8.2, 0.8 Hz, 1H), 6.27 (dd, J = 7.7, 0.9 Hz, 1H), 5.30 (s, 2H). ^{13}C NMR (101 MHz, d_6-DMSO) δ 154.5 (C), 132.1 (C), 130.6 (C), 130.1 (C), 124.1 (CH), 108.9 (CH), 97.5 (CH).

7-(Piperazin-1-yl)benzo[*d*]oxazol-2(3*H*)-one[2]

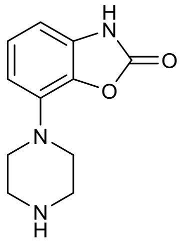

7-Aminobenzo[*d*]oxazol-2(3*H*)-one (520 mg, 3.46 mmol) was taken up in chlorobenzene (20 mL) and heated at reflux until all starting material was dissolved. In a separate flask, *bis*(2-chloroethyl)amine hydrochloride (618 mg, 3.46 mmol) was taken up in water (5 mL) and extracted into chloroform (2 × 20 mL) by the addition of 1 M NaOH (5 mL). The combined organic extracts were dried over anhydrous sodium sulfate then added directly into the hot reaction mixture and left to stir overnight at reflux. The reaction solution was then decanted from the vessel, leaving a precipitate adhered to the glassware. The vessel was washed with further chlorobenzene (10 mL) then the precipitate taken up in methanol (20 mL) and purified by flash column chromatography ($CHCl_3$:MeOH:NH_4OH, 80:19:1, v/v) to give the title compound as a pale yellow oil (381 mg, 50%).

^{1}H NMR (400 MHz, CD_3OD) δ 7.05 (dd, *J* = 8.3, 7.8 Hz, 1H), 6.68 – 6.63 (m, 2H), 3.25 – 3.19 (m, 4H), 3.04 – 2.99 (m, 4H). ^{13}C NMR (101 MHz, CD_3OD) δ 157.1 (C), 137.3 (C), 135.8 (C), 132.9 (C), 125.7 (CH), 111.4 (CH), 104.1 (CH), 50.9 (CH_2), 46.3 (CH_2).

7-(4-([1,1'-Biphenyl]-3-ylmethyl)piperazin-1-yl)benzo[*d*]oxazol-2(3*H*)-one (bifeprunox)[2]

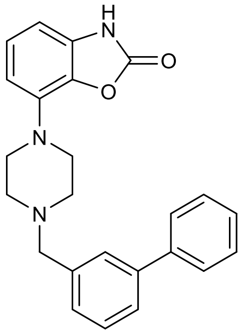

7-(Piperazin-1-yl)benzo[*d*]oxazol-2(3*H*)-one (30 mg, 137 µmol) was taken up in MeCN (10 mL) and to the yellow solution was added 3-(bromomethyl)biphenyl (34 mg, 137 µmol) and sodium iodide (4.1 mg, 27 µmol). The solution was heated at reflux for 16 h, after which point the solvent was removed *in vacuo*. The residue was then taken up in EtOAc (15 mL) and washed with 1 M K_2CO_3 (2 × 15 mL), brine (15 mL), dried over anhydrous sodium sulfate, then purified by flash column chromatography (EtOAc:Petroleum spirits, 1:1, v/v) to give the title compound as a clear oil (32 mg, 61%).

^{1}H NMR (400 MHz, $CDCl_3$) δ 9.60 (br s, 1H), 7.62 – 7.56 (m, 3H), 7.54 – 7.48 (m, 1H), 7.47 – 7.37 (m, 3H), 7.37 – 7.31 (m, 2H), 7.02 (app t, *J* = 8.1 Hz, 1H), 6.60 (dd, *J* = 7.8, 0.8 Hz, 1H), 6.58 (dd, *J* = 8.4, 0.8 Hz, 1H), 3.67 (s, 2H), 3.41 – 3.24 (m, 4H), 2.78 – 2.63 (m, 4H). ^{13}C NMR (101 MHz, $CDCl_3$) δ 155.5 (C), 141.3 (C), 141.1 (C), 138.1 (C), 136.1 (C), 134.2 (C), 130.2 (C), 128.80 (CH), 128.78 (CH), 128.3 (CH), 128.1 (CH), 127.3 (CH), 127.2 (CH), 126.1 (CH), 124.8 (CH), 110.6 (CH), 102.6

(CH), 63.1 (CH_2), 53.0 (CH_2), 49.1 (CH_2). HRMS (*m/z*): $[MH]^+$ calcd. for $C_{24}H_{23}N_3O_2$, 386.1869; found 386.1875. HPLC, t_R = 8.20 min, >95% purity (214 nm).

7-(4-Methylpiperazin-1-yl)benzo[*d*]oxazol-2(3*H*)-one (pardoprunox)[3]

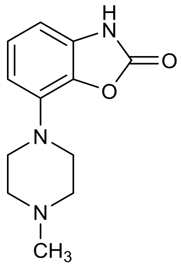

7-(Piperazin-1-yl)benzo[*d*]oxazol-2(3*H*)-one hydrochloride (250 mg, 978 µmol) was taken up in acetonitrile (3 mL) in a microwave vial. To the mixture was added *N*,*N*-diisopropylethylamine (170 µL, 978 µmol) and methyl iodide (61 µL, 978 µmol), and the brown mixture heated in the microwave for 20 min at 80 °C. The brown precipitate was removed by filtration, and washed with acetonitrile (10 mL), and the filtrate evaporated to dryness *in vacuo*. The product was then purified by flash column chromatography ($CHCl_3$:MeOH:NH_4OH, 95:5:0.1, v/v) to give the title compound as a pale yellow solid (80 mg, 35%). For further purification, the solid was taken in $CHCl_3$ (5 mL) and triturated with toluene (10 mL) to remove excess *N*,*N*-diisopropylethylamine. The solid was then collected by filtration, washed with toluene (10 mL) and dried to give the title compound as a white solid.

^{1}H NMR (400 MHz, d_6-DMSO) δ 9.58 (br s, 1H), 7.07 (t, J = 8.1 Hz, 1H), 6.76 – 6.67 (m, 2H), 3.86 – 3.38 (m, 4H), 3.28 – 2.98 (m, 4H), 2.88 (s, 3H). ^{13}C NMR (101 MHz, d_6-DMSO) δ 153.9 (C), 133.52 (C), 133.46 (C), 131.3 (C), 124.4 (CH), 110.5 (CH), 103.4 (CH), 52.3 (CH_2), 46.1 (CH_2), 42.3 (CH_3). HRMS (*m/z*): $[MH]^+$ calcd. for $C_{12}H_{15}N_3O_2$, 234.1237; found 234.1239. HPLC, t_R = 4.60 min, >95% purity (214 nm).

Supplementary References

1. Gottlieb, H., Kotlyar, V. & Nudelman, A. NMR chemical shifts of common laboratory solvents as trace impurities. *J. Org. Chem.* **62,** 7512–7515 (1997).

2. Gant, T. & Sarshar, S. 3H-Benzoxazol-2-ones as modulators of D2 receptor and/or 5-HT1A receptor and their preparation and use in the treatment of diseases. US20100119622A1 (2010).

3. Rheenen, J., Muijselaar, W. & Teunissen, H. Preparation of polymorphs of pardoprunox. US20110086862A1 (2011).
